# Seismic evidence for subduction-induced mantle flows underneath Middle America

**DOI:** 10.1038/s41467-020-15492-6

**Published:** 2020-04-29

**Authors:** Hejun Zhu, Robert J. Stern, Jidong Yang

**Affiliations:** Department of Geosciences, The University of Texas at Dallas, Dallas, TX USA

**Keywords:** Geophysics, Seismology

## Abstract

Laboratory experiments and geodynamic simulations demonstrate that poloidal- and toroidal-mode mantle flows develop around subduction zones. Here, we use a new 3-D azimuthal anisotropy model constructed by full waveform inversion, to infer deep subduction-induced mantle flows underneath Middle America. At depths shallower than 150 km, poloidal-mode flow is perpendicular to the trajectory of the Middle American Trench. From 300 to 450 km depth, return flows surround the edges of the Rivera and Atlantic slabs, while escape flows are inferred through slab windows beneath Panama and central Mexico. Furthermore, at 700 km depth, the study region is dominated by the Farallon anomaly, with fast axes perpendicular to its strike, suggesting the development of lattice-preferred orientations by substantial stress. These observations provide depth-dependent seismic anisotropy for future mantle flow simulations, and call for further investigations about the deformation mechanisms and elasticity of minerals in the transition zone and uppermost lower mantle.

## Abstract (web summary)

The motions of subducted slabs are expected to drive mantle flow around slab edges, however, evidence of deep mantle flow has so far remained elusive. Here, the authors present a Full Waveform Inversion 3-D anisotropy model which allows them to infer deep subduction-induced mantle flows underneath the Mid-Americas and the Caribbean.

## Introduction

Mapping mantle flows induced by descending oceanic lithospheres has profound implications for understanding the dynamic, thermal and chemical evolution of subduction systems^1,2^. Two end-member flow circulation patterns have been proposed based on geodynamical, geochemical and seismological observations. The first end-member suggests that the downdip movement of slabs entrains mantle flows to great depths, causing 2-D corner flows perpendicular to the trench in the mantle wedge and beneath the slabs, also known as the poloidal-mode flow^3^. The second end-member suggests that, to preserve the mass displaced by trench migration and slab rollback, 3-D return flows develop in the sub-slab region and surrounding the lateral edge of sinking slabs, also known as the toroidal-mode flow. The toroidal-mode flow typically happens for convergent plate margins with fast trench migration speeds^4,5^, and is important for controlling trench curvature^6^. To date, the behaviors of these two end-members have been extensively investigated using laboratory experiments^7,8^ and mantle flow simulations^9,10^.

Seismic anisotropy is a valuable tool to detect mantle flows in subduction zones^11,12^. Olivine and orthopyroxene, two dominant upper mantle minerals, are intrinsically anisotropic in terms of seismic wavespeeds^13,14^. The strain-induced lattice-preferred orientation (LPO) of olivine leads to the direction and polarization dependence of seismic wavespeeds^15^, which can be detected using shear wave splitting and surface wave tomography. Previous shear wave splitting studies document a trench-parallel pattern beneath the fore-arc and the subducted slab^4,16^, as well as a trench-perpendicular pattern in the back-arc mantle^3^. However, shear wave splitting measurements represent the path-integrated effects of anisotropy over the entire raypath, leading to poor vertical resolution and controversial interpretations^17^. Surface wave tomography, on the other hand, enables us to constrain depth-dependent azimuthal anisotropy by taking advantage of the dispersive characteristics of Rayleigh and Love waves^18–21^. However, its lateral resolution is typically limited to several hundreds of kilometers due to the use of long-period signals, making it difficult to resolve small-scale features, such as the toroidal-mode mantle flows in the vicinity of sinking slabs. The different lateral and depth resolutions of shear wave splitting and surface wave tomography also make it challenging to reconcile these two independent measurements^20,22^.

**Fig. 1:**
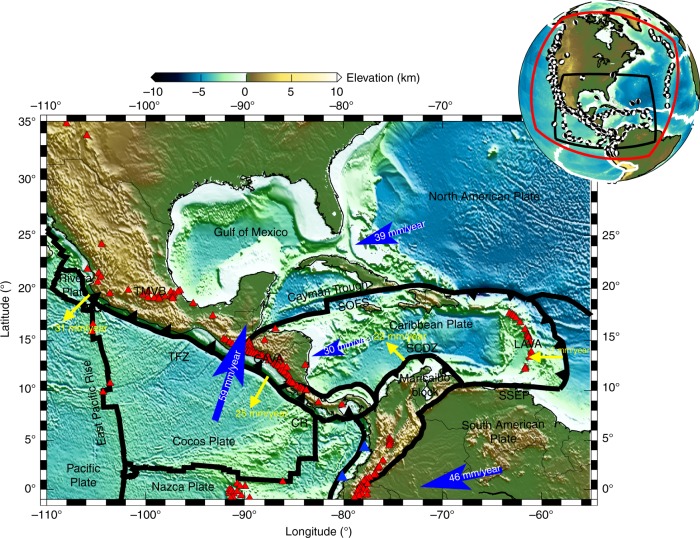
Tectonic map for the Middle American and Caribbean Subduction Zones. Black lines denote global plate boundaries with triangles showing subduction directions^87^. Red triangles are volcanoes (www.ngdc.noaa.gov/hazard). Blue arrows denote plate motion directions^58^, and yellow arrows show trench migration velocities^55^, all with the Pacific hotspot reference frame. The top right insert shows the location of the study region, with the red box showing the inversion domain. Beach balls denote the moment tensor solutions for earthquakes used in the inversion (www.globalcmt.org). Abbreviations for tectonic structures are: CAVA Central American Volcanic Arc, CR Cocos Ridge, EGG El Gordo Graben, LAVA Lesser Antilles Volcanic Arc, SCDZ Southern Caribbean Deformation Zone, SOFS Septentrional-Oriente Fault System, SSEP San Sebastian-El Pilar Fault System, TFZ Tehuantepec Fracture Zone, TMVB Tran-Mexico Volcanic Belt.

In this study, we characterize detailed, depth-dependent seismic anisotropy for the Middle American and Caribbean subduction systems using full waveform inversion^23–25^. The Middle American Subduction Zone (MASZ) is a 2700 km long, active convergent margin, stretching from Mexico to Costa Rica (Fig. 1), which involves the subduction of the young Rivera and Cocos Plates underneath the North American and Caribbean Plates. Both the Rivera and Cocos Plates are the remnants of the ancient Farallon Plate, which broke into several fragments in the Oligocene^26^. Currently, the 10 Myr oceanic crust of the Rivera Plate is subducting underneath the Jalisco Block of Mexico at a speed of 30 mm/yr. Along the strike of the Middle American Trench (MAT), the convergence rate of the Cocos Plate relative to the overriding Caribbean Plate increases from 50 mm/yr in southern Mexico to 90 mm/yr offshore Nicaragua and Costa Rica^27^.

As forming in the Cretaceous, the Caribbean Plate has undergone complicated interactions with the North American, South American and Farallon Plates^28^. It is now bounded to the north by a 1200 km east-west orientated, left-lateral Septentrional-Oriente Fault System, and to the south by another >1000 km long, right-lateral fault, the San Sebastian-El Pilar Fault System. To the west, it is confined by the eastward descending Cocos Plate, and to the east by the westward sinking Atlantic Oceanic lithosphere to form the Lesser Antilles Volcanic Arc (LAVA). The transition from the subduction of the Atlantic slab to transform motion along the San Sebastian-El Pilar Fault System has been suggested as an example of a Slab-Transform Edge Propagator (STEP) fault^29^. Two tectonic reconstructions have been proposed to explain the origin of the Caribbean Plate: the Pacific^30^ and intra-Americas origin^31^, placing this plate to the west and east of the Farallon Plate in the Cretaceous, respectively. The arcuate-shaped Antilles Subduction Zone is still an enigma in Middle America, which includes the Greater Antilles in the north underneath Hispaniola and Puerto Rico, and the Lesser Antilles in the southeast (Fig. 1). The shallow portion of the subducting slab can be directly inferred from the Wadati-Benioff zone^32^. In the lower mantle, the slabs underneath the Caribbean Plate merge with the Cocos Plate into the prominent fast Farallon anomaly, which has been detected in global tomography models^33,34^.

The Middle American and Caribbean Subduction Zones are ideal locations to investigate the dynamics of subduction systems because of strong variations in the slab morphology^35^, fast trench migration speeds (20–30 mm/yr)^5^, the concave-shaped LAVA, as well as heterogeneous geochemical observations in the Trans-Mexico Volcanic Belt (TMVB)^36^ and Central American Volcanic Arc (CAVA)^37^. All these features indicate complex mantle flows and subduction processes underneath Middle America, which are explored by using a new azimuthal anisotropy model US_32_ in this study.

## Results

### 3-D azimuthal anisotropy model US_32_

With the framework of full waveform inversion, we combine three-component short-period (15–40 s) body waves with long-period (25–100 s) surface waves to simultaneously constrain deep and shallow structures underneath North and Middle America. The current azimuthal anisotropy model, named US_32_, is constrained by using 180 earthquakes and 4516 seismographic stations (Supplementary Fig. 2), cumulating in 586,185 frequency-dependent phase measurements. 1579 stations from the USArray Transportable Array, and other permanent and temporary arrays in North and Middle America are used in the inversion, including the Mapping the Rivera Subduction Zone (MARS), Tomography Under Costa Rica and Nicaragua (TUCAN), Meso-America Subduction Experiment (MASE), Veracruz-Oaxaca Subduction (VEOX), etc. Supplementary Table 1 gives the numbers of stations in major contributing arrays (with stations > 30) located in Middle America and used for constructing model U*S*_32_. The current model is the result of 32 preconditioned conjugate gradient iterations. The first 22 iterations are utilized to constrain radially anisotropic model parameters, including horizontally propagating, vertically (*β*_v_) and horizontally (*β*_h_) polarized shear wavespeeds^38^. The last ten iterations are used to further constrain radially (*L* and *N*) and azimuthally anisotropic (*G*_c_ and *G*_s_) model parameters. These four model parameters are simultaneously updated using the Fréchet derivatives calculated by the adjoint state method, details for constructing the model can be found in Zhu et al.^39^ and methods.

**Fig. 2:**
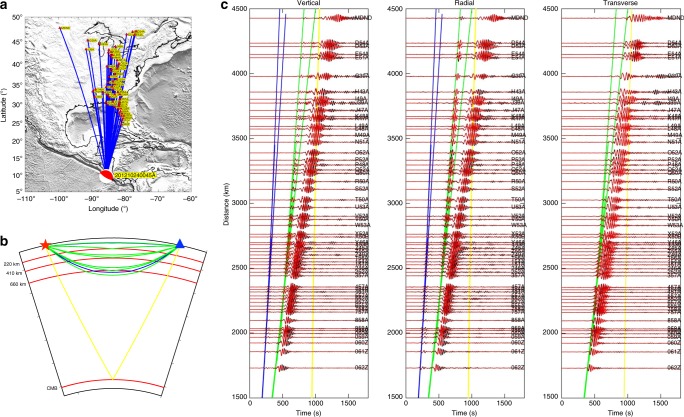
Comparisons of observed and predicted seismograms from model US_32_. **a** The locations of 1 earthquake and 58 stations from the USArray deployed in the eastern U.S. This Mw 6.0 earthquake occurred at a depth of 18.3 km underneath Costa Rica on October 24th, 2012 (CMTSOLUTION_201210240045A). **b** The theoretical ray paths of P (blue), S (green), ScS (yellow) phases in the spherical symmetric PREM model^88^, with the epicentral distance equal to 25°. Red lines denote the 220, 410, 660-km discontinuities and the core mantle boundary. **c** Compares three-component, observed (black) and predicted (red) seismograms for TA stations shown in **a**. From left to right are vertical, radial and transverse components, respectively. Blue, green and yellow lines denote the theoretical arrival times for P, S and ScS phases from the PREM model. Both observed and predicted seismograms are bandpass filtered from 25 to 100 s.

Figure 2 demonstrates the capability of full waveform inversion to match three-component observed (black) and predicted (red) seismograms for a shallow earthquake that occurred in 2012 underneath Costa Rica, both observations and predictions are bandpass filtered from 25 to 100 s. High-quality data from the high-density USArray enables us to capture complex, triplicated body waves passing through the upper mantle discontinues, and offers us opportunities to harness these signals for delineating detailed structures near these phase transition boundaries. In Fig. 2, three-component body waves, as well as Rayleigh and Love waves are matched very well, suggesting that the mantle structures underneath Middle America are well constrained in the current model US_32_. More waveform fittings can be found in Supplementary Note 1, and comparisons with previous tomographic studies can be found in Supplementary Note 6.

### Resolution analysis

**Fig. 3:**
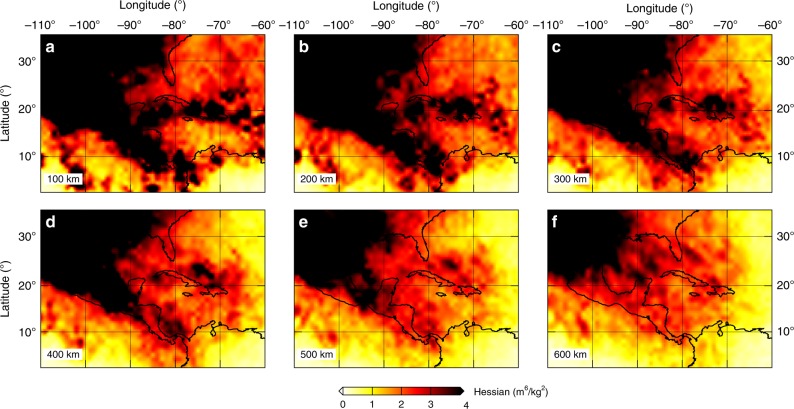
The distribution of the approximated diagonal Hessian for the study region. **a**–**f** Results at depths ranging from 100 to 600 km. Warm color represents good illumination in comparison to cool color.

Analyzing resolution or uncertainty of full waveform inversion is important and challenging, considering computational costs for performing iterative inversion^40–43^. Here, we use the approximated diagonal Hessian and point-spread functions (PSFs) to analyze illumination, resolution, and more importantly tradeoffs among different model parameters for several key features discussed in the following sections. Figure 3 shows the horizontal distribution of the approximated diagonal Hessian underneath Middle America at depths ranging from 100 to 600 km, which is a good indicator for the ray density coverage^44^. Owing to the configuration of stations and earthquakes (Supplementary Fig. 2), the illumination for North America, the MAT and the Lesser Antilles is good at all depth ranges. In contrast, the illumination for the central Caribbean is not as good as other regions, especially at greater depths.

**Fig. 4:**
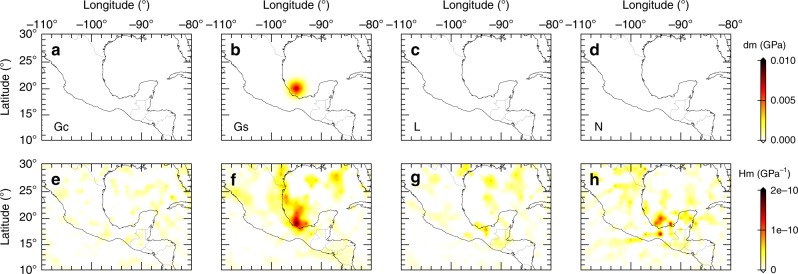
One point-spread function test at 350 km offshore Mexico. **a**–**d** The input Gaussian perturbations for *G*_c_, *G*_s_, *L* and *N*, respectively. **e**–**h** The PSFs with respect to these four model parameters.

Figure 4 presents one PSF test around the Rivera slab offshore Mexico, a location with interesting features for discussing toroidal-mode mantle flows. In this calculation, a Gaussian anomaly with a half width of 120 km is added to *G*_s_ at a depth of 350 km, and three other model parameters (*G*_c_, *L* and *N*) are held unchanged. Results for this PSF test suggest that there are good constraints for the fast axis orientations at this location, which depend on the ratio between *G*_c_ and *G*_s_. For instance, there is limited leakage from *G*_s_ to other three model parameters, and the resulting PSF for *G*_s_ perturbation is well focused. More PSF tests can be found in Supplementary Note 2. These PSFs give us confidence about the distributions of wavespeeds and anisotropic fabrics at these locations.

### Shallow structures

**Fig. 5:**
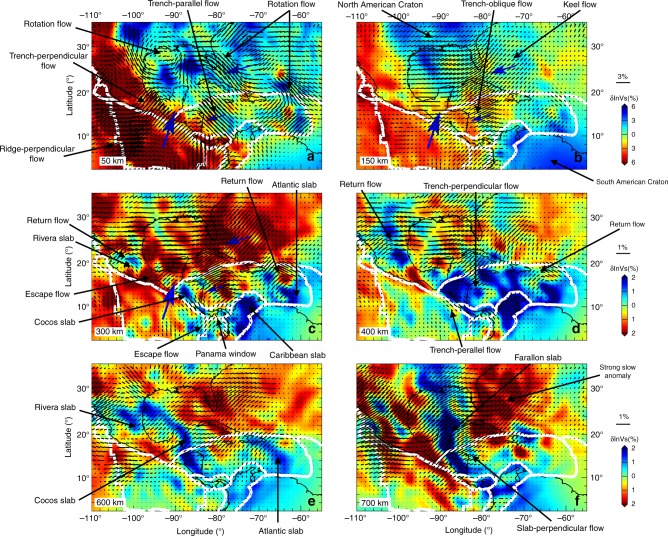
Horizontal cross sections of relative perturbations in isotropic shear wavespeed and azimuthal anisotropy in model US_32_. **a**–**f** Results at depths ranging from 50 to 700 km. 1D reference model STW105^89^ is used to calculate the relative wavespeed perturbations. The direction and magnitude of the fast axes are given by the orientation and length of the black bars. Key features discussed in the main text are highlighted by *arrows*, and white lines denote global plate boundaries^87^. Blue arrows at depths ranging from 50 to 300 km denote plate motion directions^58^.

**Fig. 6:**
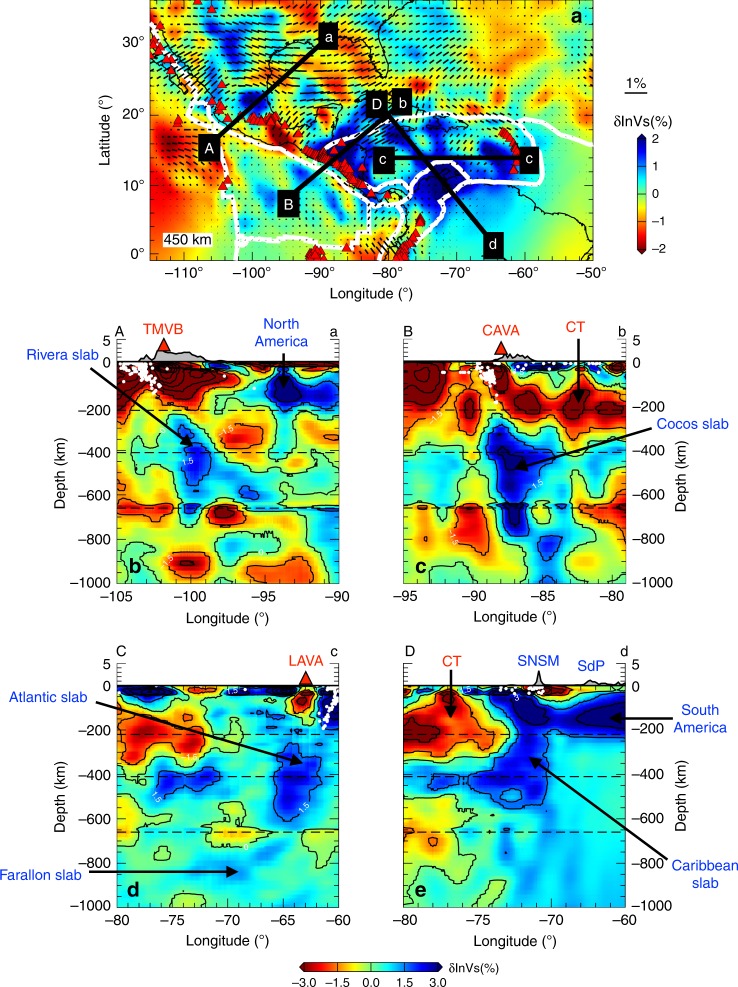
Vertical cross sections of relative perturbations in isotropic shear wavespeed for the Middle American and Caribbean Subduction Zones. **a** Shear wavespeed anomalies and fast directions at a depth of 450 km, as well as the locations of four vertical cross sections shown in **b**–**e**. The dashed black lines in these cross sections denote the 220, 410 and 660-km discontinuities. Earthquakes with magnitude >5 are shown by white circles, and red triangles represent volcanoes. CAVA Central American Volcanic Arc, CT Cayman Trough, SNSM Sierra Nevada de Santa Marta, LAVA Lesser Antilles Volcanic Arc, SdP Serranía del Perijá, TMVB Trans-Mexico Volcanic Belt.

**Fig. 7:**
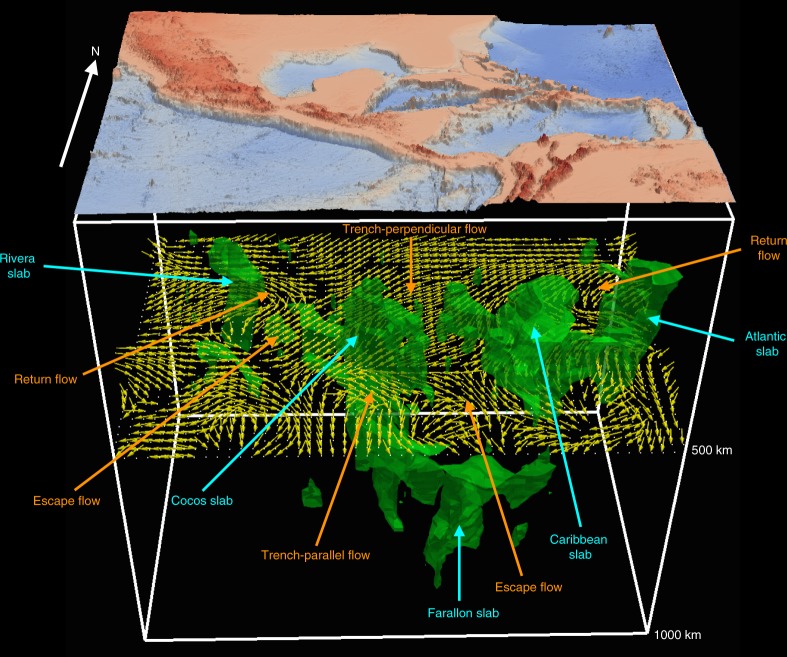
3-D iso-surface representations of fast wavespeed anomalies with magnitude >1.5% (green bodies), and fast axes (yellow arrows) at a depth of 500 km in model US_32_. Topographic variations are superimposed on top of the 3-D iso-surface bodies. Fast anomalies shallower than 250 km, mainly the North and South American lithospheres, are clipped for better visualization of sinking slabs. Cyan arrows are used to highlight slab features, whereas orange arrows are used to denote mantle flow fields.

**Fig. 8:**
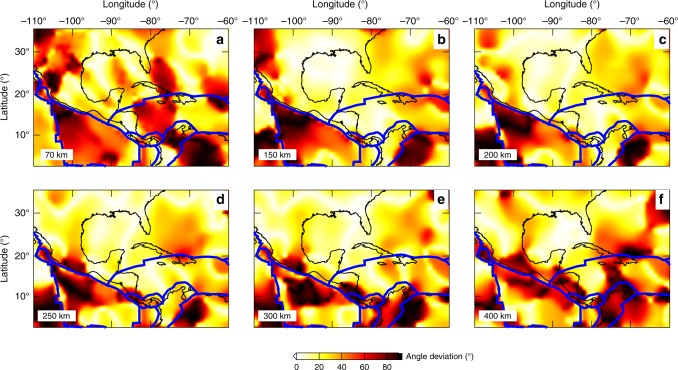
Angle differences between the fast axis orientations in model US_32_ and the plate motion model NUVEL-1A^58^ with the hotspot reference frame. **a**–**f** Results at depths ranging from 70 to 400 km, with warm color representing large angle differences. Blue lines denote the plate boundaries^87^.

**Fig. 9:**
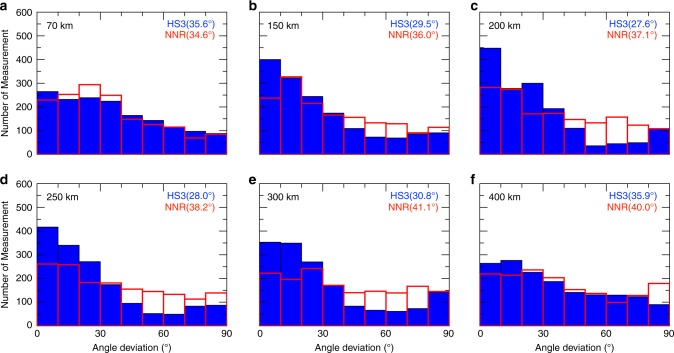
The histograms of angle differences between the fast axis orientations in model US_32_ and the plate motion model NUVEL-1A^58^. **a**–**f** Results at depths ranging from 70 to 400 km. Blue and red histograms are results for the hotspot and no-net rotation reference frames, respectively. Numbers in the brackets to the right corner of each histogram denote mean angle differences between these measurements.

Figure 5 presents horizontal cross sections of isotropic shear wavespeed anomalies and fast axis orientations in model US_32_ at depths ranging from 50 to 700 km (more slices can be found in Supplementary Note 3). The detailed morphologies of the descending Rivera, Cocos, Caribbean and Atlantic slabs can be examined in the vertical cross sections of Fig. 6 and 3-D iso-surface visualization in Fig. 7. At depths shallower than 200 km, anisotropic signatures in model US_32_ follow wavespeed heterogeneities, tectonic provinces as well as global plate motion directions (Figs. 8 and 9). For instance, the fast directions are mostly perpendicular to the strike of the MAT, reflecting 2-D subduction-induced corner flows at shallow depths^3^. The East Pacific Rise is characterized by prominent slow wavespeed anomalies, and the anisotropic fabrics are aligned perpendicular to the ridge axis, in agreement with seafloor spreading between the Pacific and Cocos Plates. Furthermore, underneath the western Atlantic Ocean, the fast directions are running parallel to the U.S. coastline, suggesting the development of asthenospheric flows surrounding the deep keel of the North American lithosphere^45^, which reaches to around 250 km (Supplementary Fig. 12). Another potential interpretation of this pattern could be mantle flows affected by the subduction of the Nazca and Farallon slabs as demonstrated in geodynamic flow simulations^46,47^. At depths in excess of 300 km, model US_32_ exhibits several fast wavespeed anomalies associated with the descending Rivera, Cocos, Atlantic and Caribbean Plates, as well as complicated anisotropic fabrics in their vicinity.

### Subduction-induced mantle flows

The narrow Rivera slab is imaged dipping ~40° northeast underneath the Jalisco Block in Mexico (profile A-a in Fig. 6). It appears that the Rivera slab is detached from the surface, despite the occurrence of Mw > 5 earthquakes at depths shallower than 150 km, which is consistent with global tomography results^48^. Its mantle wedge is characterized by slow wavespeed anomalies, suggesting the ascent of hot asthenosphere from 400 km towards the TMVB, which might explain uplift and low Bouguer gravity anomalies (~−100 mgal) in this region (Supplementary Fig. 1). The subducted Rivera and Cocos slabs are separated by a slab window, filled with pronounced slow anomalies (<−2%) from 300 km down to the transition zone around 12–18°*N* (Fig. 5), in agreement with previous body and surface wave tomographic results^49,50^. Furthermore, the fast axes are revealed following the lateral edge of the Rivera slab, and moving across the slab window (Figs. 5 and 7). This pattern reflects the development of return flows surrounding the narrow (~200 km), fast rollback (~31 mm/yr) Rivera slab, as well as escape flows moving across slab windows, owing to large pressure gradients created by slab fragmentations as demonstrated in previous physical and numerical experiments^6,7^. The return flows and slab detachment might contribute to the observed mixed subduction-related calc-alkaline rocks with intraplate-like lavas and adakites along the TMVB^36^, as well as the unusual oblique angle (16°) between the MAT and TMVB.

In profile B-b (Fig. 6) and 3-D visualization (Fig. 7), the Cocos slab plunges ~60° down to the transition zone underneath Nicaragua and Costa Rica. Its northern boundary is truncated sharply along the extension of the Septentrional-Oriente Fault System (Fig. 5). Flat subduction of the Cocos Plate might exist underneath central Mexico (Supplementary Fig. 14), however, it is still challenging to detect thin, horizontal contrasts at shallow depths using seismic tomography because of its limited vertical resolution. The observation of different dip angles of the Rivera and Cocos slabs along the strike of the MAT is consistent with previous studies from seismicity distribution^35^, local tomography^49^ and receiver functions^51^. The steeper dip of the Cocos slab can be attributed to its older age (23 Myr) and therefore greater density. Similar to the Rivera slab, the Cocos slab is also detached from the surface, with the mantle wedge filled with strong slow seismic anomalies connecting to the CAVA and Cayman Trough. Moreover, the trench-parallel anisotropic pattern is observed in the sub-slab region of the Cocos slab at depths ranging from 300 to 450 km (Fig. 5). There is a sharp transition in the mantle wedge from the trench-parallel pattern at 50 km to the trench-perpendicular pattern at greater depths. These complex, depth-dependent anisotropic fabrics suggest the development of toroidal-mode flows in the sub-slab region, in response to fast slab rollback (25 mm/yr) of the MAT^5^. Toroidal-mode flows are forced to tilt toward shallower depths in the mantle wedge due to fast slab rollback, and interfere with the corner flows entrained by the downdip motion of the Cocos slab, in agreement with 3-D geodynamic flow simulations^9^. Furthermore, the fast axes in model US_32_ run across the Panama slab window^28^ between the Cocos and Nazca Plates at 300–400 km depth. This observation supports the hypothesis about the subduction of the Cocos-Nazca Ridge and Galápagos hotspot track in this region^28^, and offering an explanation about the occurrence of Neogene alkaline and arc-tholeiitic volcanism in Panama^52^.

Underneath the LAVA, the Atlantic lithosphere sinks toward the west with ~50° dip down to the transition zone (Figs. 6C-c and 7). In contrast to the Rivera and Cocos slabs, the Wadati-Benioff zone beneath the LAVA reflects subduction from the surface down to 200 km, with a slow anomaly imaged in the mantle wedge underneath the LAVA. In Fig. 5, there are two fast bodies at 300–400 km depth, one underneath Puerto Rico and the other underneath the southern LAVA, which are separated by a slab window around 16–18°N. Previous P wave tomography studies report a similar feature, which was attributed to the subducted North American-South American plate boundary^28,53^. Similar to the anisotropic fabrics observed around the Rivera slab, the fast directions surround the lateral edge of the descending Atlantic lithosphere, and move across the slab window (Figs. 5 and 7). This pattern suggests the development of return flows warping around the narrow, rapidly advancing (17 mm/yr) Atlantic slab, which pushes materials within the mantle wedge and induces flows around its edge. The current model fails to delineate east-west oriented anisotropic fabrics along the San Sebastian-El Pilar Fault System as demonstrated in shear wave splitting^54^, suggesting limited resolution due to the lack of stations at this location (Supplementary Fig. 2).

In profile D-d (Fig. 6), the Caribbean slab dips southeast underneath the Southern Caribbean Deformation Zone and Maracaibo Block. Its dip increases from 30° at depths shallower than 200 km to around 90° at greater depths, which might be affected by the presence of the thick (~250 km), craton-like overriding South American Plate. To the northwest, the Cayman Trough is underlain by a triangular slow anomaly (<−1.5%) down to 300 km, reflecting strong deformation owing to the relative motion and pull-apart between the North American and Caribbean Plates along the Septentrional Fault System since the Eocene^28^. By pushing mantle materials in the sub-slab region, the fast northwestward rollback of the Caribbean slab (22 mm/yr)^55^ might also contribute to the observed northeast-southwest oriented flow field underneath the Caribbean Plate at depths ranging from 300 to 500 km (Fig. 5).

At 600 km, the fast wavespeed anomalies discussed above merge into a continuous, arcuate-shaped feature surrounding the Gulf of Mexico and Caribbean, which then turns to a north-south oriented fast Farallon anomaly at 700 km^34^, stretching from the southern U.S. to South America (Fig. 5). Strong slow anomalies are observed to the west and east of the Farallon slab, which might reflect water entrained down to the transition zone by subduction of the Farallon Plate since the Mesozoic. In comparison to the upper mantle and transition zone, the fast orientations turn perpendicular to the strike of the Farallon anomaly, reflecting poloidal-mode mantle flows in the uppermost lower mantle. This flow field might be driven by strains produced when the Farallon slab penetrated the 660-km discontinuity to enter the lower mantle. This process results in large stresses due to a significant viscosity jump across the 660-km discontinuity^56^, and might produce detectable strain-induced seismic anisotropy.

## Discussion

### Comparisons with plate motion data

Debayle et al.^57^ concluded that surface wave azimuthal anisotropy models correlate well with global plate motion models^58,59^ for regions with a relatively simple tectonic history, such as oceanic plates. However, for regions with a complicated tectonic history, such as continents, their correlation is much poorer. Figure 8 presents the angle differences between the fast axis orientations in model US_32_ for the study region with the global plate motion model NUVEL-1A^58^ at depths ranging from 70 to 400 km. Both hotspot (Fig. 8) and no-net rotation (Supplementary Fig. 17) reference frames are used for these comparisons. Figure 9 shows the histograms for these angle differences at different depths. Overall, the current seismic model fits plate motion results better for the hotspot reference than the no-net rotation reference at all depths. For instance, from 150 to 300 km, the mean angle differences for the hotspot reference are <30°, whereas these numbers are >35° for the no-net rotation reference. It appears that the fast axis orientations in the current model fit plate motions with the hotspot reference frame better for the North Atlantic and Caribbean. In contrast for the Cocos Plate, the no-net rotation frame fits seismic measurements better. Major differences come from regions with complicated fast axis orientations, especially in the vicinity of subducting slabs, which are not included in large-scale plate motion results.

### Implications for mantle flow simulations and deformation mechanisms in the deep Earth

LPO of anisotropic mineral aggregates deformed by dislocation creep is the major cause of seismic anisotropy. Experiments demonstrate that the major minerals within the mantle transition zone and the uppermost lower mantle, such as wadsleyite and bridgmanite, might also be intrinsically anisotropic^60,61^. Diffusion creep or superplasticity is considered as the dominant deformation mechanism in the deep mantle^62^, and this prevents the development of LPO. Therefore, the existence and nature of seismic anisotropy within the transition zone and lower mantle are still highly debated. A recent study reported strong radial anisotropy (up to 2%) in the vicinity of subducting slabs down to 1000–1200 km^63^, which raises questions about the deformation mechanisms in the deep Earth. In addition, a number of previous surface wave tomography^64,65^ and shear wave splitting^66,67^ studies speculated about whether or not azimuthal anisotropy exists in the deep mantle, especially around subducted slabs. Anisotropic signatures (with magnitude around 1%) observed at greater depths in this study provide new seismic evidence for the development of complicated mantle flows induced by subduction systems with fast trench migration speeds and strong variations in slab morphology, such as tearing and detachment. All these seismic observations suggest that dislocation creep might have a more important role in the deep Earth than we thought before. In addition, all interpretations in this article assume that we have A-, C- and E-type LPO for olivine aggregates, other types of LPO, such as type-B fabric affected by water distributions, may change current interpretations^14,68^, especially for the mantle wedge regions.

Furthermore, depth-dependent azimuthal anisotropy observed in this study offers more constraints for investigating the physics of subduction, such as using mantle flow simulations^9,10^ and physical experiments^7,8^. Most recent mantle flow simulations allow us to construct geodynamic models to fit different geophysical and geological observations, such as shear wave splitting^54,69^ and plate motions^58^. However, shear wave splitting represents the integrated anisotropic effects over the entire depth range^17^. Therefore, it is a non-unique solution to match splitting measurements through LPO calculations^70^ and flow simulations. The depth-dependent azimuthal anisotropy patterns delineated in this study provide future opportunities to better constrain 3-D deep mantle flows and investigate physical processes through geodynamic modeling.

### Misfit function

The details of constructing model US_32_ can be found in Zhu et al.^39^ Here, we summarize the key components of this study. The spectral-element method^71,72^ is used to solve the anisotropic/anelastic wave equation, and compute three-component synthetic seismograms. Each forward simulation takes 45 minutes using 144 CPU cores, therefore, in total, it took 1.87 million CPU hours to construct the current model. Frequency-dependent phase measurements for three-component short-period (15–40 s) body waves and long-period (25–100 s) surface waves are combined to simultaneously constrain deep and shallow structures^73^. No crustal correction^74,75^ is required in the inversion owing to the simultaneous update for the crustal and mantle structures. A multi-scale inversion strategy^76^, i.e., starting with long-period signals for long-wavelength updates and gradually incorporating short-period signals to improve resolution, is utilized to mitigate the cycle skipping problem^77^. The total misfit function includes six categories: P-SV body waves on vertical and radial components, and SH body waves on transverse component; Rayleigh surface waves on vertical and radial components, and Love surface waves on transverse component. A multi-taper technique^78^ is employed to calculate frequency-dependent phase discrepancies between observed and predicted seismograms within automatically selected windows from FLEXWIN^79^. The total misfit is defined as follows

1 $$J = \frac{1}{2}\mathop {\sum }\limits_{i = 1}^{N_{\mathrm{c}}} w_{\mathrm{c}}\mathop {\sum }\limits_{m = 1}^{N_m} {\int} {w_m\left[ {\frac{{{\mathrm{\Delta }}\tau _m(\omega )}}{{\sigma _m^\phi (\omega )}}} \right]^2{\mathrm{d}}\omega ,}$$

where *N*_c_ denotes the total number of categories, here *N*_c_ = 6 for three-component body and surface wave contributions. *w*_c_ is the weighting factor used to balance the magnitudes of each individual category. *w*_*m*_ represents the weighting factor used for the multi-taper measurements. Functions Δ*τ*_*m*_ (*ω*) and $\sigma _{m}^\phi (\omega )$ represent the frequency-dependent phase anomalies and their associated uncertainties, respectively.

### Model parameterization

There are 13 independent model parameters in a typical tomographic study for simultaneously constraining radial and azimuthal anisotropy^18,80^. However, due to the non-uniqueness of geophysical inverse problems and limited sensitivity of seismic data, not all of them can be well resolved in real applications. Previous theoretical studies have suggested that Rayleigh waves are mostly sensitive to 2*θ* azimuthal dependent coefficients *G*_c_ and *G*_s_^81,82^, whereas Love waves have weak sensitivity to *E*_c_ and *E*_s_. Here subscripts c and s stand for the cosine and sine terms, respectively, and *θ* denotes the local azimuth. In this study, we combine three-component surface and body waves to simultaneously constrain four model parameters: two radially anisotropic parameters *L* and *N*, and two azimuthally anisotropic parameters *G*_c_ and *G*_s_^39^.

Thus, the relative perturbation of the total misfit, *δJ*, can be expressed as the following volume integral

2 $$\delta J = {\smallint_{{\!\!\!}V}} {{K}}_L\delta L + {{K}}_N\delta N + {{K}}_{G_{\mathrm{c}}}\delta G_{\mathrm{c}} + {{K}}_{G_{\mathrm{s}}}\delta G_{\mathrm{s}}{\mathrm{d}}V$$

These four model parameters are related with the fourth-order elastic tensor *C*_*ij*_ as

3 $$L 	= \,\frac{1}{2}\left( {C_{44} + C_{55}} \right), \\ N 	= \,\frac{1}{8}\left( {C_{11} + C_{22} - 2C_{12} + 4C_{66}} \right), \\ G_{\mathrm{c}} 	= \,\frac{1}{2}\left( {C_{55} - C_{44}} \right), \\ G_{\mathrm{s}} 	= \,- C_{45}.$$

### Misfit gradients

The misfit gradients with respect to the four model parameters *L*, *N*, *G*_c_ and *G*_s_ can be calculated as follows^83,84^

4 $${{K}}_L 	= \, {{K}}_{{\mathrm{c}}_{44}} + {{K}}_{{\mathrm{c}}_{55}}, \\ {{K}}_N 	= \, {{K}}_{{\mathrm{c}}_{66}} - 2{{K}}_{{\mathrm{c}}_{12}}, \\ {{K}}_{G_{\mathrm{c}}} 	= \, {{K}}_{{\mathrm{c}}_{55}} - {{K}}_{{\mathrm{c}}_{44}}, \\ {{K}}_{G_{\mathrm{s}}} 	= \, - {{K}}_{{\mathrm{c}}_{45}}.$$

Here the primary gradient *K*_*cij*_ can be computed via the adjoint state method^85^ as

5 $$K_{c_{ij}} = - \smallint_{{\!\!}0}^{T} \varepsilon _i^\dagger (x,T - t)c_{ij}(x)\varepsilon _j(x,t){\mathrm{d}}t,$$

where *ε*_*j*_ and $\varepsilon _{i}^{\dagger}$ are the elements of the strain tensors for the forward and adjoint wavefields, respectively.

A preconditioned conjugate gradient method^86^ is used to iteratively update the above four model parameters. The final isotropic shear wavespeed *V*_s_ and radially anisotropic parameter *ξ* can be calculated from *L* and *N* as

6 $$V_{\mathrm{s}} 	= \sqrt {(2L + N)/3\rho } , \\ \xi 	= N/L.$$

With the distributions of *G*_c_ and *G*_s_, the peak-to-peak anisotropic strength *G*_0_ and fast axis direction *ϕ* are computed via

7 $$G_0 	= \sqrt {G_{\mathrm{s}}^2 + G_{\mathrm{c}}^2} , \\ \phi 	= \frac{1}{2}{\mathrm{arctan}}(G_{\mathrm{s}}/G_{\mathrm{c}}).$$

## Supplementary information

Supplementary Information

Description of Additional Supplementary Files

Supplementary Data 1

Supplementary Movie 1

Supplementary Movie 2

Supplementary Movie 3

## Data Availability

All the continuous seismic data are collected from the Incorporated Research Institutions for Seismology (IRIS) Data Management Center (http://www.iris.edu/ds/nodes/dmc/). The digital model for US_32_ can be downloaded from the attached data file.

## Code availability

The open source spectral-element software package SPECFEM3D_GLOBE and the seismic measurement software package FLEXWIN used in this study are freely available for download via the Computational Infrastructure for Geodynamics (CIG; geodynamics.org). This is UTD Geoscience contribution number 1357.
