# Supplementary material for: Seismic evidence for subduction-induced mantle flows underneath Middle America
Source: Nat Commun. 2020 Apr 29;11:2075. doi: 10.1038/s41467-020-15492-6 (PMC7190827; doi:10.1038/s41467-020-15492-6)
Supplement: Supplementary file 1 — Supplementary Information [file 41467_2020_15492_MOESM1_ESM.pdf]

Supplementary Information for "Seismic evidence for subduction-induced mantle flows underneath Middle America"

Hejun Zhu,[1*], Robert J. Stern[1] and Jidong Yang[1]

[1]Department of Geosciences, The University of Texas at Dallas, Texas, USA
[*]To whom correspondence should be addressed; E-mail: hejun.zhu@utdallas.edu.

Supplementary Note 1 More waveform comparisons

Supplementary Figures 3 –8 compare three-component, observed (black) and predicted (red) seismograms calculated from the current model US_{32} for six earthquakes that occurred in Middle America from 2004 to 2013. The frequency band used for these waveform comparisons ranges from 25 to 100 seconds. Supplementary Figures 3 –5 present waveform comparisons for local temporary arrays deployed in the study region. For instance, event CMTSOLUTION_200609101456A (Supplementary Figure 3) was a Mw 5.9 earthquake occurred at a depth of 32.2 km underneath the Gulf of Mexico. Seismograms recorded by 31 MARS stations on the northwestern coast of Mexico are aligned as a function of epicentral distances. For this small aperture array, waveform fittings for both body and Rayleigh waves are quite good for the vertical component. However, the data quality is low for the radial and transverse components, therefore, their fittings are poorer in comparison to the vertical component. These comparisons suggest that there is a good constraint for the Rivera subduction system underneath northwestern Mexico.

The second event CMTSOLUTION_200601232050A (Supplementary Figure 4) was a Mw 6.1 earthquake occurred at a depth of 21.8 km underneath the west coast of Colombia. Seismograms from 59 stations deployed in Nicaragua, Costa Rica, Venezuela and the Lesser Antilles islands are compared, giving us a wide azimuthal coverage with epicentral distances ranging from 500 to 2,000 km. The fittings for both body and Rayleigh waves on the vertical and radial components are good, suggesting a good constraint for the Panama window and the Atlantic subduction system. Furthermore, event CMTSOLUTION_091804A (Supplementary Figure 5) was a Mw 5.7 earthquake occurred at a depth of 15.6 km underneath the North Atlantic Ocean. 30 stations from the TUCAN deployment are utilized for comparisons. For these long distance, narrow azimuth records, observed and predicted waveforms are matched very well for the vertical component, similar to the previous two examples. Some records for the radial and transverse components have poorer data quality, leading to poor waveform fittings. These comparisons allow us to verify the illuminations of the current model US_{32} for the Cayman Trough and the Cocos subduction system underneath Nicaragua and Costa Rica.

Moreover, Supplementary Figures 6–8 present waveform comparisons for three earthquakes recorded by stations from the USArray located in the western, central and eastern U.S. respectively. The first event CMTSOLUTION_200703172243A (Supplementary Figure 6) was a Mw 6.0 shallow, normal faulting earthquake that occurred at a depth of 5.4 km south of Panama. 70 TA stations, deployed in the western U.S. in 2007, are used for waveform comparisons. All three-component data are matched very well, including P, S, Rayleigh and Love waves, even with the long propagation distances ranging from 4,300 to 6,600 km. By taking advantage of the high-density and high-quality USArray deployment, it is very promising to utilize some specific reflection and refraction body wave signals, such as triplicated phases

from upper mantle discontinuities, to further constrain detailed structures inside the Earth. Furthermore, event CMTSOLUTION_201001191423A (Supplementary Figure 7) was a Mw 5.7 earthquake that occurred at a depth of 12.5 km offshore Cuba, and recorded by 65 TA stations deployed in the central U.S. in 2010. This is a left-lateral, strike slip event occurred along the Septentrional-Oriente Fault. Again, there are very good waveform fittings for P, S, Rayleigh and Love waves for all three-component seismograms, suggesting a good constraint for the mantle structure underneath the Gulf of Mexico and central U.S. Finally, event CMT-SOLUTION_201302091416 (Supplementary Figure 8) was a Mw 6.7 deep earthquake that occurred at a depth of 150.5 km underneath western Colombia. Data from 66 TA stations deployed in the eastern U.S. in 2013 are collected for this comparison. In contrast to the previous two examples associated with shallow earthquakes, surface waves are not well developed for this deep event. The current model US_{32} enables us to match complicated three-component waveforms for body waves, including depth phases, providing a good constraint for the Farallon subduction system in the uppermost lower mantle underneath the eastern U.S., Gulf of Mexico and Caribbean. These body waves will be utilized in the next step to further delineate detailed, fine-scale mantle structures underneath North and Middle America.

Supplementary Note 2 More resolution analysis

Supplementary Figure 9 presents another PSF test around the Atlantic slab underneath the LAVA at a depth of 350 km, accompanying Figure 4 in the main text. These PSFs suggest that there are good constraints for the fast axis orientations at this location. Furthermore, Supplementary Figures 10 and 11 show two additional PSF tests at a depth of 240 km underneath the Rivera and Cocos Subduction Zones. Instead of perturbing G_s as Supplementary Figure 9, here we add perturbations for G_c and L to illustrate tradeoffs for other model parameters. As demonstrated in Supplementary Figure 10, there is leakage from G_c to L since both of them are related to the combination of C_{44} and C_{55}. But overall, we are able to resolve anomalies at these shallow depths, giving us confidence about the detachment features for the Rivera and Cocos slabs.

Supplementary Note 3 More cross sections for model US_{32}

Supplementary Figures 12–15 present more horizontal and vertical cross sections for model US_{32}, accompanying Figures 5 and 6 in the main text. These allow us to further illustrate the morphologies of the Rivera, Cocos, Atlantic and Caribbean slabs, as well as complicated mantle flows surrounding these sinking oceanic lithospheres.

Supplementary Figure 14 presents several vertical cross sections along and perpendicular to the strike of the MAT, allowing us to better investigate the changing dip angles for the Rivera and Cocos slabs along the trench, as observed from previous seismicity studies [1].

Previous receiver function and seismicity studies reported the flat subduction of the Cocos slab underneath central Mexico [1, 2, 3, 4]. As demonstrated in cross section C-c, to date, it is still challenging to use seismic tomography to delineate this thin, subhorizontal subduction, due to the lack of vertical resolution and smearing of nearly vertically incident body waves at shallow depths. In cross section C-c, there is a strong slow wavespeed anomaly ascending from the mantle transition zone towards the Earth's surface in the sub-slab region. Whether this reflects a mantle plume or not requires further investigations. Cross sections E-e to H-h are utilized to examine the morphology of the Cocos slab underneath Central America.

Supplementary Figure 15 employs a number of vertical cross sections to further examine the behavior of the descending Atlantic and Caribbean slabs underneath the Caribbean Sea. Cross sections A-a to C-c delineate the westward dip of the Atlantic slab underneath the LAVA. In cross section E-e, there is a fast wavespeed anomaly at depths shallower than 200 km underneath Puerto Rica, which might indicate the subduction of the North American slab [5]. A slow wavespeed anomaly is imaged at depths shallower than 100 km in cross section D-d, separating two fast anomalies to the north and south at 14–18^oN. This observation is similar to the latest P wave local tomography [6], which was interpreted as the boundary between the North and South American Plates.

Supplementary Note 4 Comparisons with SKS measurements

We use the following equations [7] to compute predicted SKS splitting measurements from our tomographic model, assuming weak anisotropy with a horizontal symmetry axis.

$$\Delta t = \left[\int_0^R \frac{G_s(\theta,\phi,r)}{v_s^0(r)L^0(r)}\mathrm{d}r\right]^2 + \left[\int_0^R \frac{G_c(\theta,\phi,r)}{v_s^0(r)L^0(r)}\mathrm{d}r\right]^2 \quad , \tag{S1}$$

$$\tan 2\Phi = \frac{\int_0^R \frac{G_s(\theta,\phi,r)}{v_s^0(r)L^0(r)}\mathrm{d}r}{\int_0^R \frac{G_c(\theta,\phi,r)}{v_s^0(r)L^0(r)}\mathrm{d}r} \quad . \tag{S2}$$

where Δt is the predicted station-averaged SKS delay time and Φ is the fast axis direction. v_s^0 and L^0 are the elastic parameters from the 1-D spherical symmetric reference model, such as STW105 [8] used in this study. G_c and G_s are the 3-D azimuthally anisotropic parameters in the inverted model, i.e., US_{32}. R is the Earth's radius. This comparison is challenging due to different lateral and depth resolutions of SKS splitting measurements and surface wave tomography [7, 9].

Supplementary Figure 16 compares predicted (red) and actual SKS (blue) measurements [9] for the study region. Here, observed SKS measurements for northern Mexico, Nicaragua and Costa Rica come from the MARS [10] and TUCAN [11] experiments. For the entire study region, the angle differences between observed and predicted SKS measurements are quite

large (with the mean value around 37.5^o in Supplementary Figure 16B), and also the predicted SKS amplitudes are smaller than actual measurements, with the mean splitting time difference around -0.91 s, suggesting that the anisotropic strengths in the current model US_{32} might be underestimated, especially at greater depths, even three-component body waves have been utilized for constructing the model.

To better understand these angle differences, the entire study domain is subdivided into three regions. Region 1 mainly includes the southern U.S. and northwest Mexico. Region 2 encompasses Nicaragua and Costa Rica, and region 3 contains the Lesser Antilles and northern South America. Overall, the fitting for region 1 is better than the other two regions in terms of angle differences, for instance, the mean angle difference is 34.5^o, less than 37.8^o and 39.0^o for regions 2 and 3. The current tomography model fails to predict the trench-parallel SKS splitting pattern in Nicaragua and Costa Rica [11, 12]. But as demonstrated in Supplementary Figure 12, at a depth of 50 km, there is a strong trench-parallel pattern offshore Nicaragua and Costa Rica. The reason for the discrepancy at this location requires further investigations. In addition, the current model fails to predict the west-east splitting pattern along the San Sebastian-El Pilar Fault System as reported in SKS measurements [13], probably due to the limited constraint for this location.

Supplementary Note 5 Comparisons with stress state data

The global stress state project measures maximum horizontal compressional stress fields [14, 15]. More than 60% of the current database come from earthquake focal mechanism solutions, other measurements are derived from borehole breakouts, drilling induced fractures at shallow depths, etc [16]. Supplementary Figure 18A compares the fast axis orientations in model US_{32} (blue) at a depth of 50 km with the global stress orientations (red) for the study region. For some regions, there are good agreements for these two independent measurements. For instance, both of them illustrate the trench-perpendicular pattern along the MAT, a northwest-southeast orientation underneath the Maricaibo Block and northern Mexico. However, there are other disagreements, such as along the Cayman Trough and around the LAVA. Supplementary Figure 18B presents the distribution of angle differences between anisotropy and maximum stress orientations. Around 60% measurements have angle differences less than 40°. Other places with angle differences greater than 40° might suggest that these two measurements reflect anisotropy at different depths, depending on different stress indicators used at individual sites.

Supplementary Note 6 Comparisons with previous studies

Supplementary Figure 19 compares model US_{32} with two global P wave tomography models: UU-P07 [17] and LLNL [18], at depths ranging from 300 to 700 km. Similar to the consistency

of global wavespeed tomographic models [19, 20, 21], strong correlations are observed for these three models in spite of using different datasets and inversion strategies in their developments. For instance, at 300 km, all three models reveal fast wavespeed fragments associated with the descending Rivera slab in northern Mexico, the Cocos slab in Central America and the Atlantic slab underneath the LAVA. The western Atlantic, Gulf of Mexico and Caribbean are filled with prominent slow wavespeed anomalies. Most these features persist down to a depth of 500 km. Large differences exist at a depth of 600 km, for instance, a continuous fast anomaly from the Gulf of Mexico to Panama is observed in model US_{32}, which is absent in the other two models, although model LLNL also involves the sinking Rivera slab offshore Mexico. The Panama window exists in all three models at 600 km. In the uppermost lower mantle (700 km), the Farallon slab dominates all three models from the southern U.S. to South America with a north-south orientation. Strong agreements exist between model US_{32} and UU-P07 at this depth, even for the prominent slow wavespeed anomalies in the vicinity of the Farallon slab underneath the Atlantic, Mexico and southern U.S. Overall, there is a good consistency among these three wavespeed models for the study region, confirming our interpretation about the morphology of the descending Rivera, Cocos, Atlantic and Caribbean slabs.

Furthermore, there are a number of azimuthal anisotropy models at both global and continental scales, which can be used to compare with the current model. Supplementary Figure 20 compares US_{32} with three global models: SL2016 [22], DR2013 [23], YB2013 [24], and one continental-scale model YR2011 [25], at depths of 100, 200 and 300 km. These global models are based on fundamental mode and overtone surface wave dispersions. Model YR2011 is derived based on the joint inversion of surface wave waveforms and SKS splitting measurements [26, 25]. Similar to the conclusions in [22], in comparison to the agreement in wavespeed heterogeneities as observed in Supplementary Figure 19. Currently, the agreement for azimuthally anisotropic variations for the study region is still quite poor, which requires future studies to verify these solutions using waveform comparisons or other measurements. Consistent features might include the trench-perpendicular pattern along the strike of the MAT in all three models, and keel flows at 300 km underneath the Atlantic Ocean and Gulf of Mexico in models US_{32} and YR2011.

Network codes	XT	TO	XI	ZD	XF	YO	TR	ZA	VE	YZ	PR	Y7
Numbers of stations	224	146	97	52	51	49	46	45	42	36	35	30

Supplementary Table 1: Numbers of stations for major network contributions (>30) in the current dataset, deployed in Middle America from 2003 to 2013. The experiment names of these networks can be found in www.fdsn.org/networks.

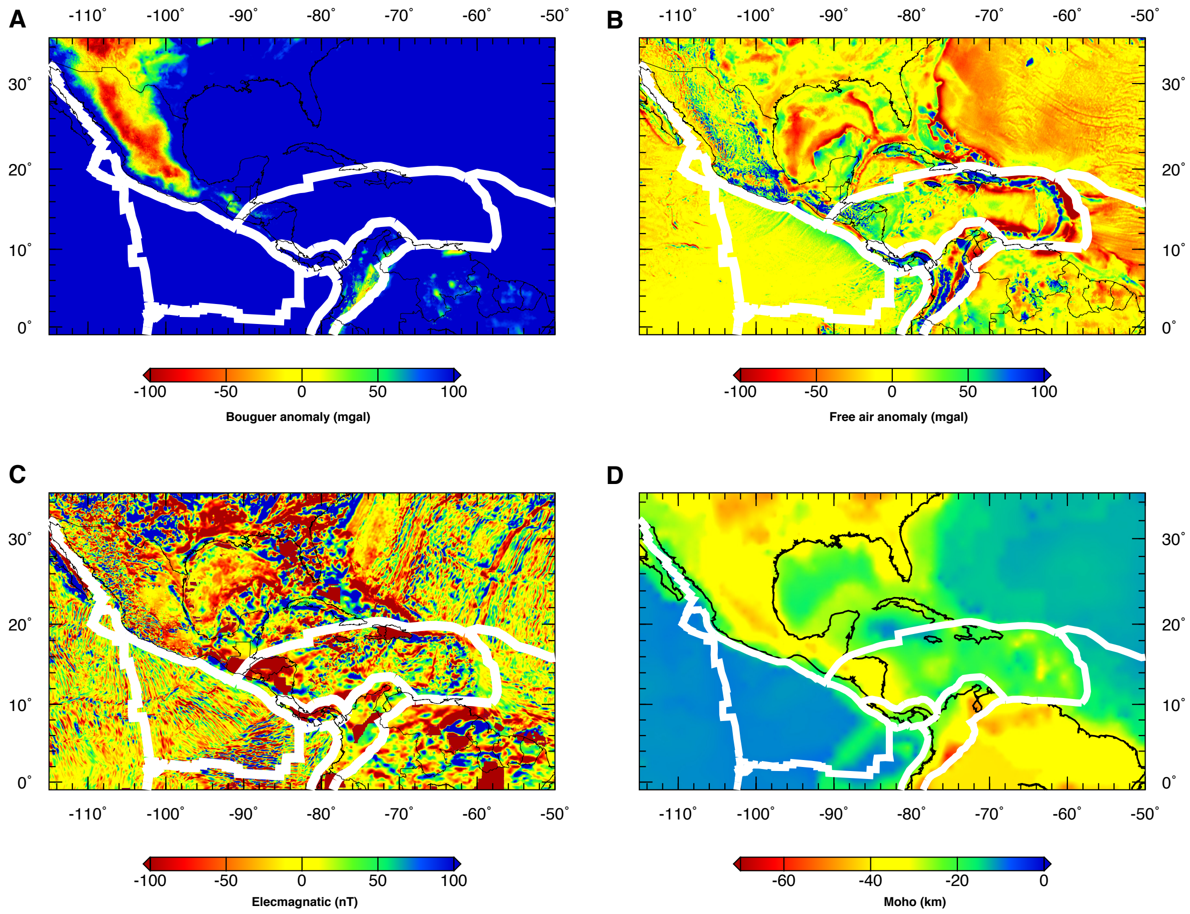

Supplementary Figure 1: Gravity, electromagnetic anomalies and the Moho depths for the study region. Bouguer (A) and free air (B) gravity anomalies are collected from model WGM2012 (http://bgi.omp.obs-mip.fr/data-products/Grids-and-models/wgm2012). Electromagnetic anomalies (C) are collected from EMAG2 (https://www.ngdc.noaa.gov/geomag/emag2.html). The Moho depths (D) come from model CRUST 1.0 [27].

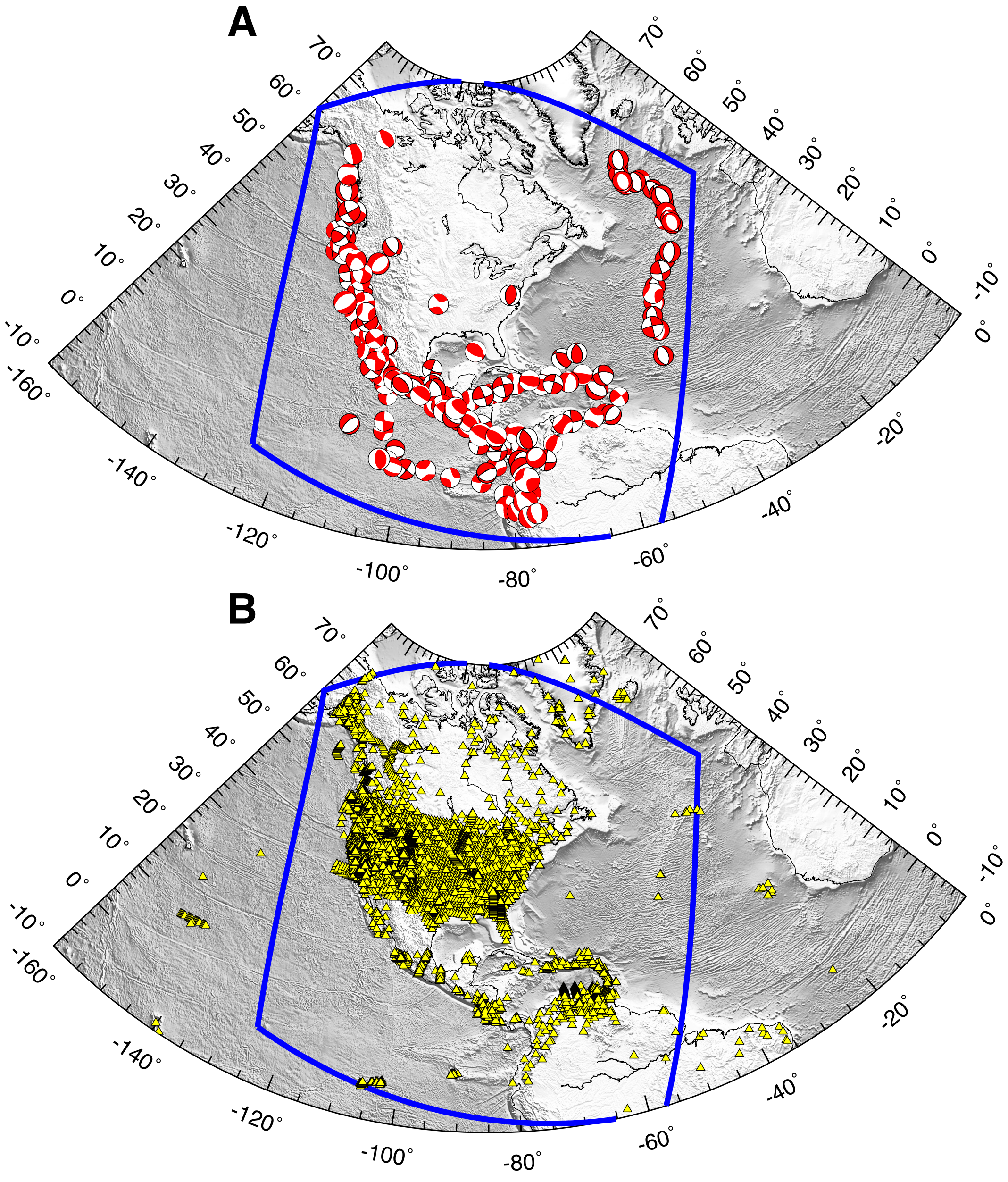

Supplementary Figure 2: Distributions of earthquakes (A) and seismographic stations (B) used for the construction of the current tomographic model US_{32}. 180 regional earthquakes occurred from 2003 to 2013 are used to illuminate the study region. Initial source parameters, such as locations and moment tensor solutions, are collected from the global CMT catalog (www.globalcmt.org). The moment magnitudes of these events range from 4.5 to 6.5. Most events have depths shallower than 30 km, with half durations ranging from 1 to 4 s [28]. The blue lines in panels A and B denote the spectral-element simulation domain. 4,516 seismographic stations are used in the inversion, among them, 1,579 stations come from the USArray Transportable Array. Other arrays deployed between 2003 and 2013 are also incorporated in the dataset.

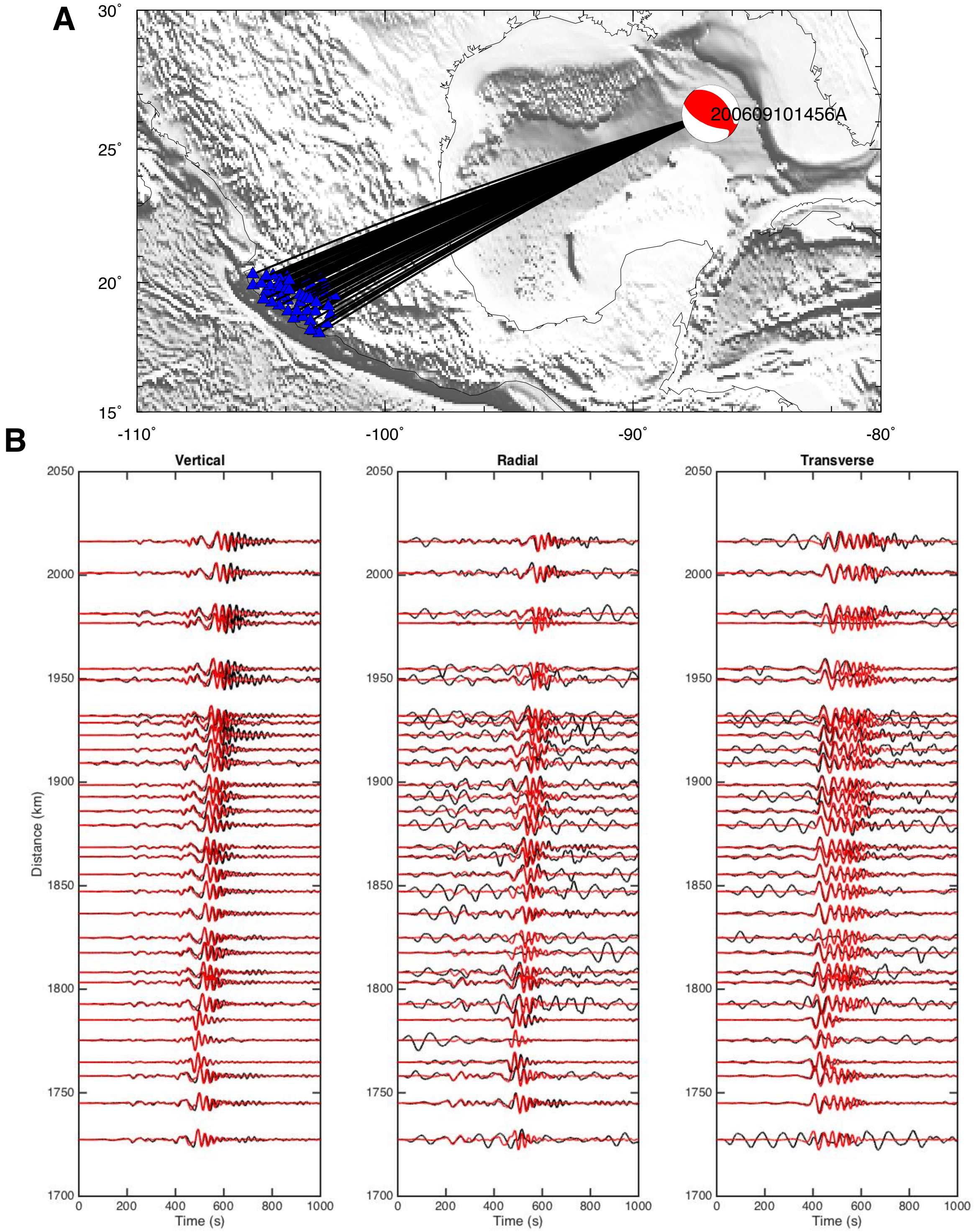

Supplementary Figure 3: Comparisons of three-component observed (black) and predicted (red) seismograms from model US_{32} for an earthquake occurred underneath the Gulf of Mexico in 2006 (CMTSOLUTION_200609101456A). Panel A shows the location of earthquake and stations from the MARS deployment. Panel B compares observed and predicted seismograms for vertical, radial and transverse components (from left to right).

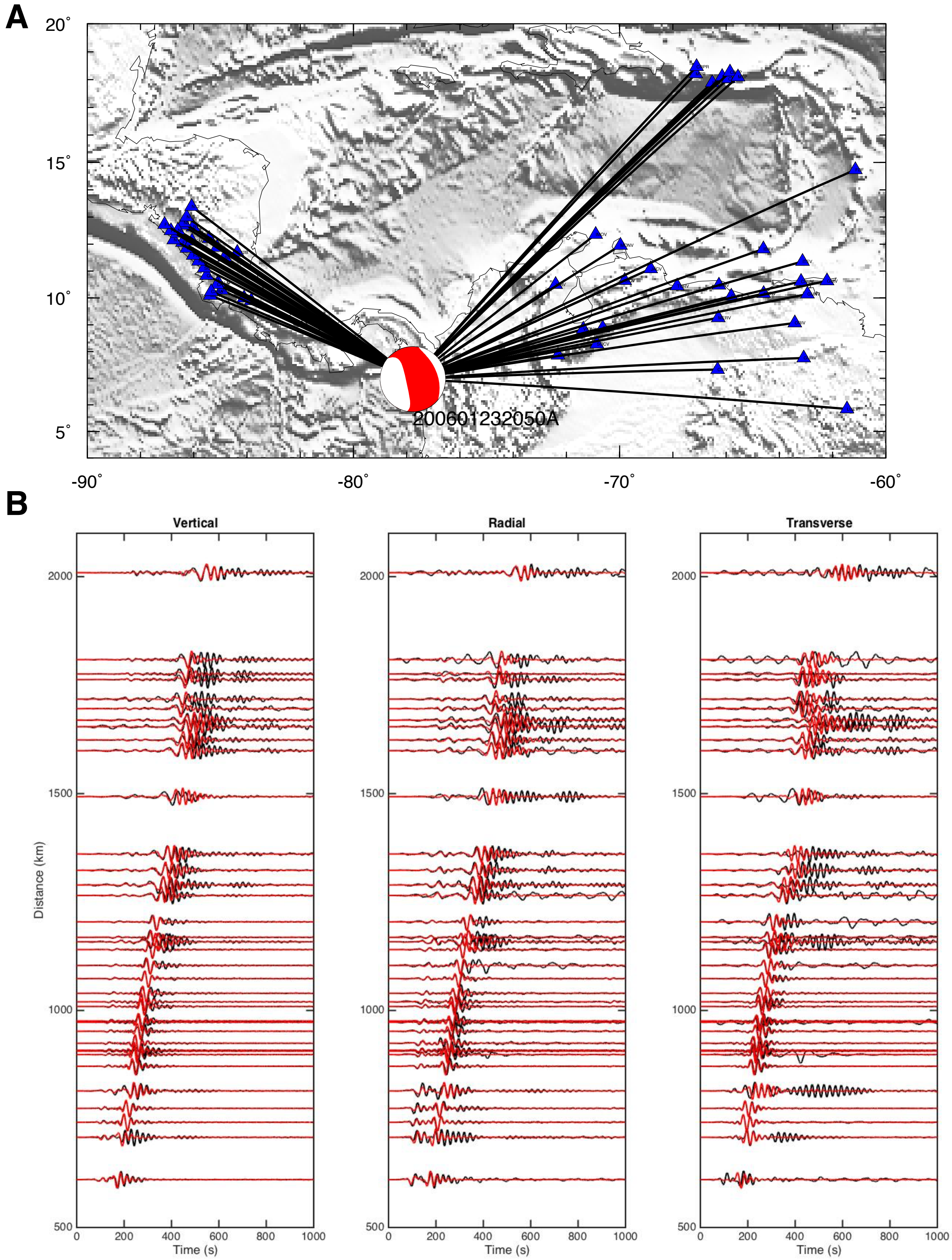

Supplementary Figure 4: Comparisons of three-component observed (black) and predicted (red) seismograms for an earthquake occurred underneath Panama in 2006 (CMTSOLUTION_200601232050A). The same setting as Supplementary Figure 3.

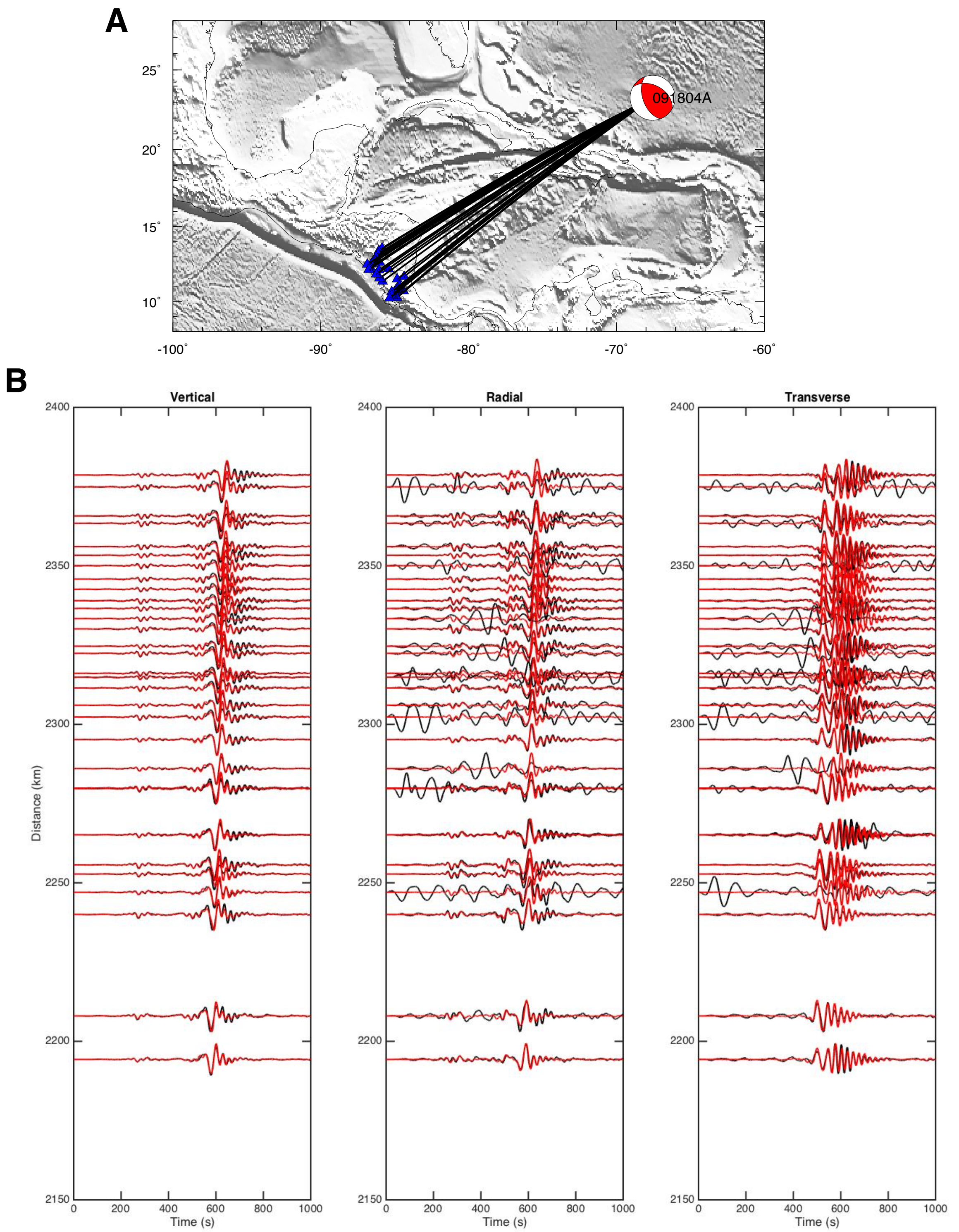

Supplementary Figure 5: Comparisons of three-component observed (black) and predicted (red) seismograms for an earthquake occurred underneath the western Atlantic Ocean in 2004 (CMTSOLUTION_091804A). The same setting as Supplementary Figure 3.

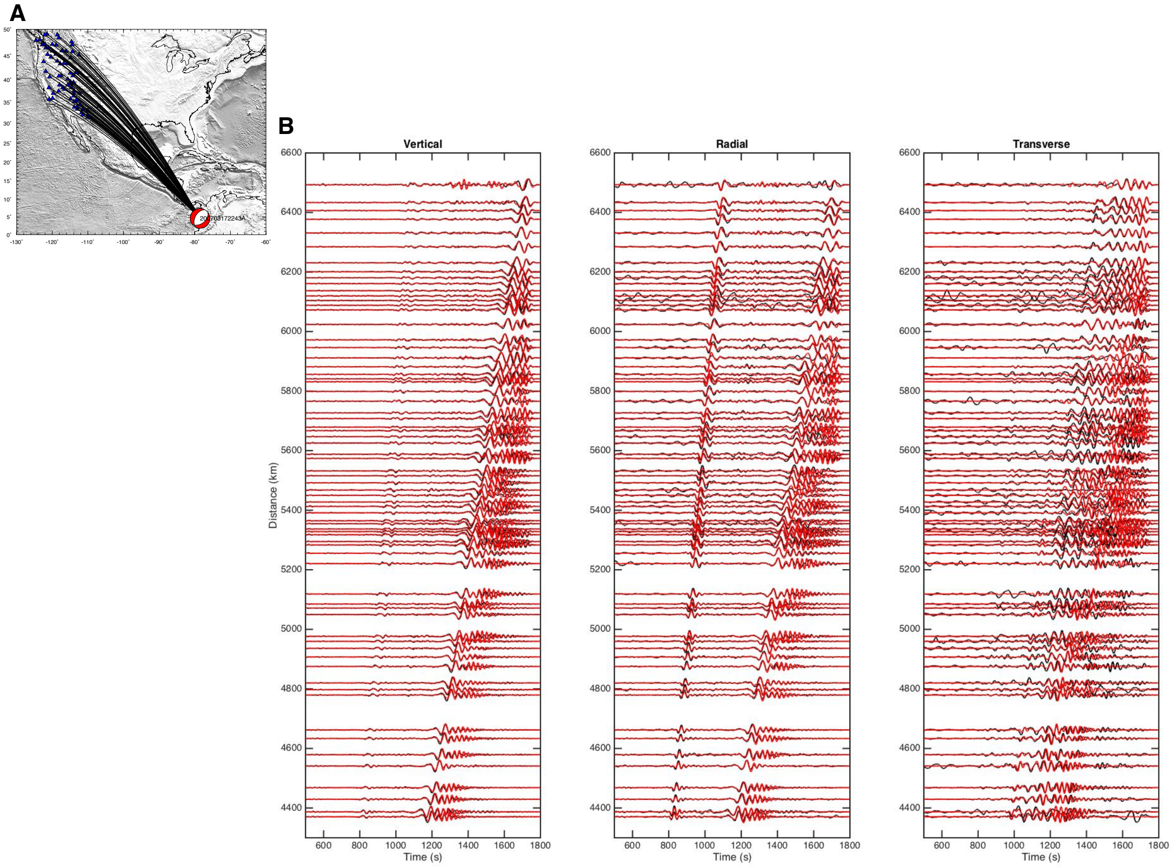

Supplementary Figure 6: Comparisons of three-component observed (black) and predicted (red) seismograms for an earthquake occurred underneath Panama in 2007 (CMTSOLUTION_200703172243A). The same setting as Supplementary Figure 3.

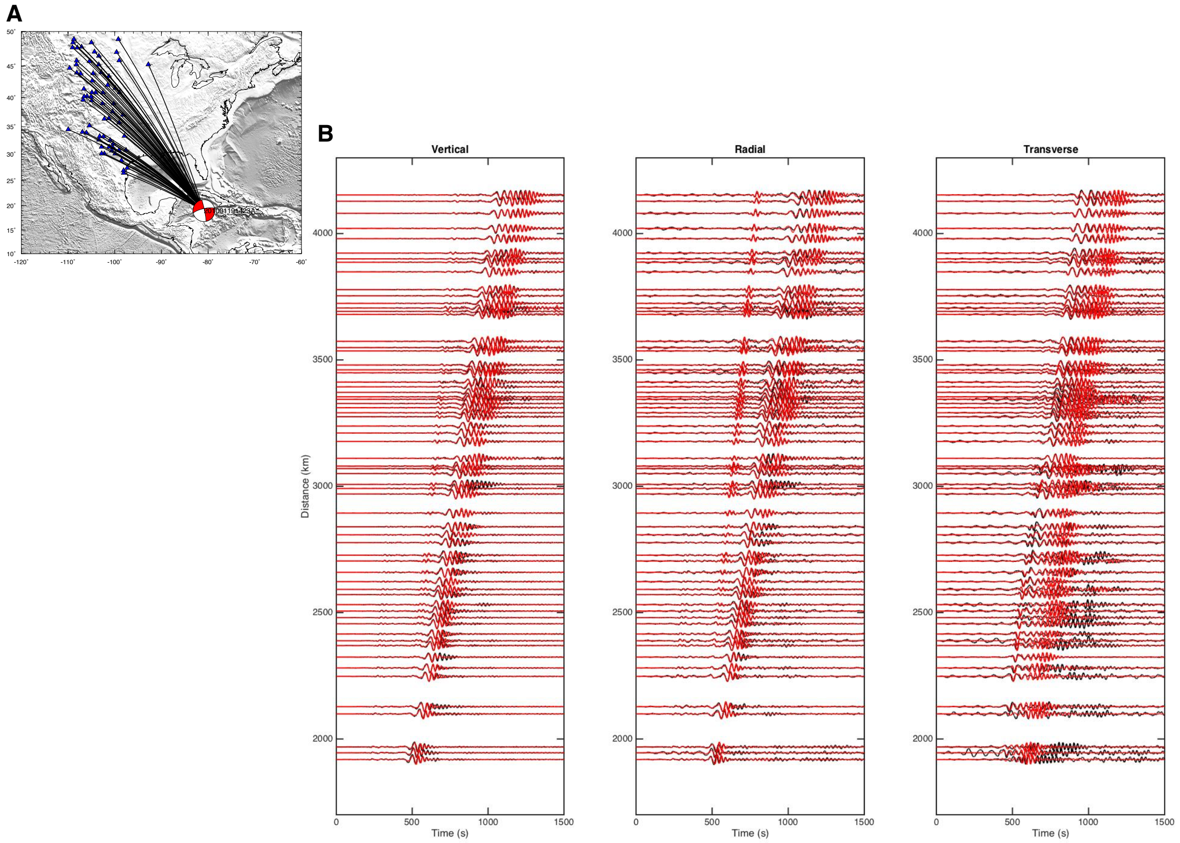

Supplementary Figure 7: Comparisons of three-component observed (black) and predicted (red) seismograms for an earthquake occurred offshore Cuba in 2010 (CMTSOLUTION_201001191423A). The same setting as Supplementary Figure 3.

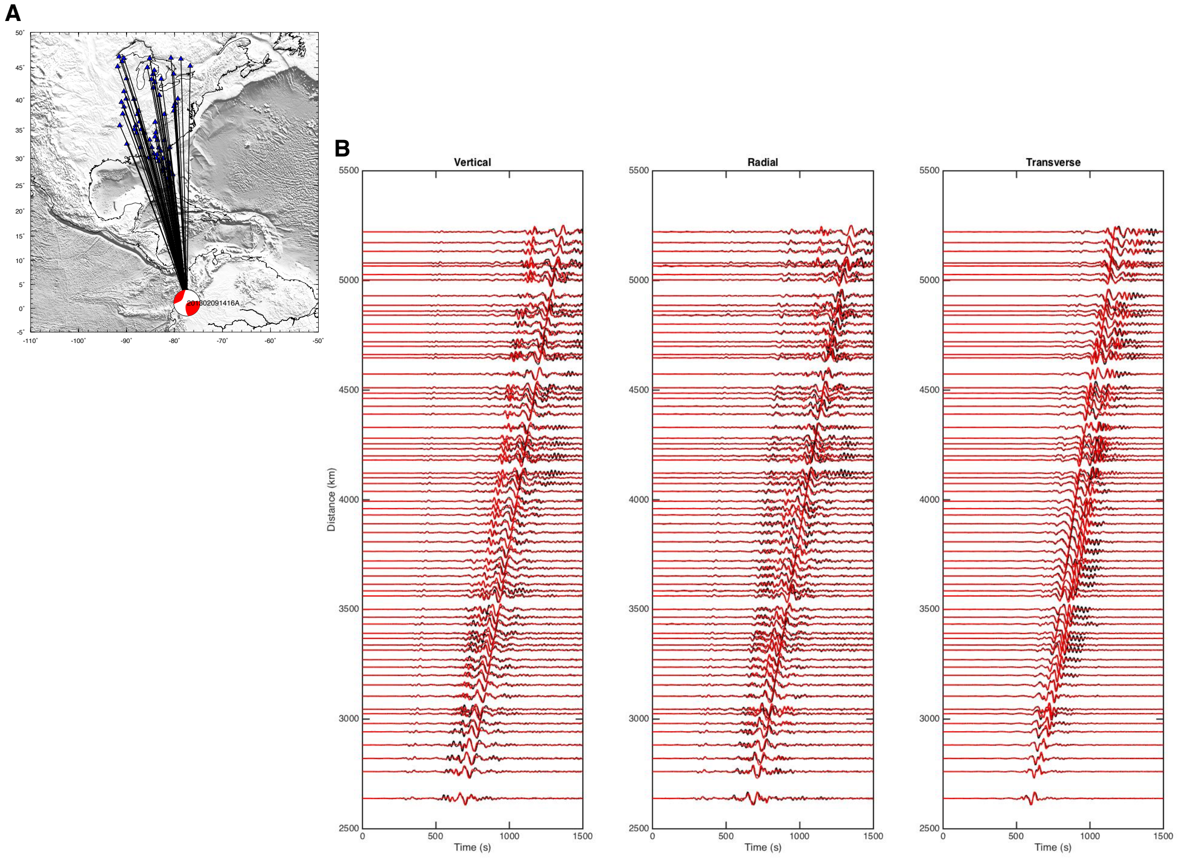

Supplementary Figure 8: Comparisons of three-component observed (black) and predicted (red) seismograms for an earthquake occurred underneath western Colombia in 2013 (CMT-SOLUTION_201302091416A). The same setting as Supplementary Figure 3.

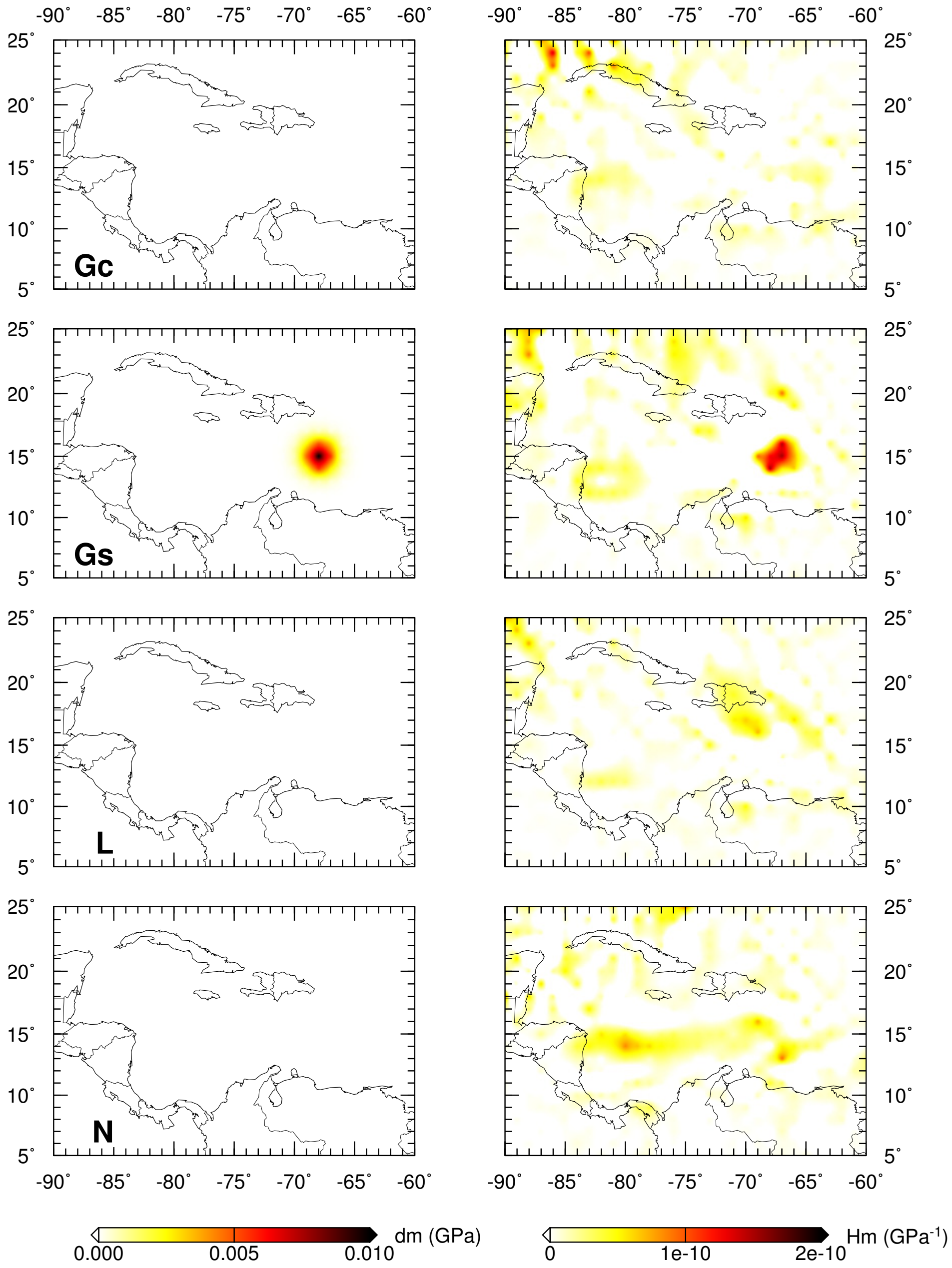

Supplementary Figure 9: The second point-spread function test at 350 km beneath the Lesser Antilles island arc. The left panel (from top to bottom) shows the input Gaussian perturbations for G_c, G_s, L and N, respectively. The right panel shows the PSFs with respect to these four model parameters.

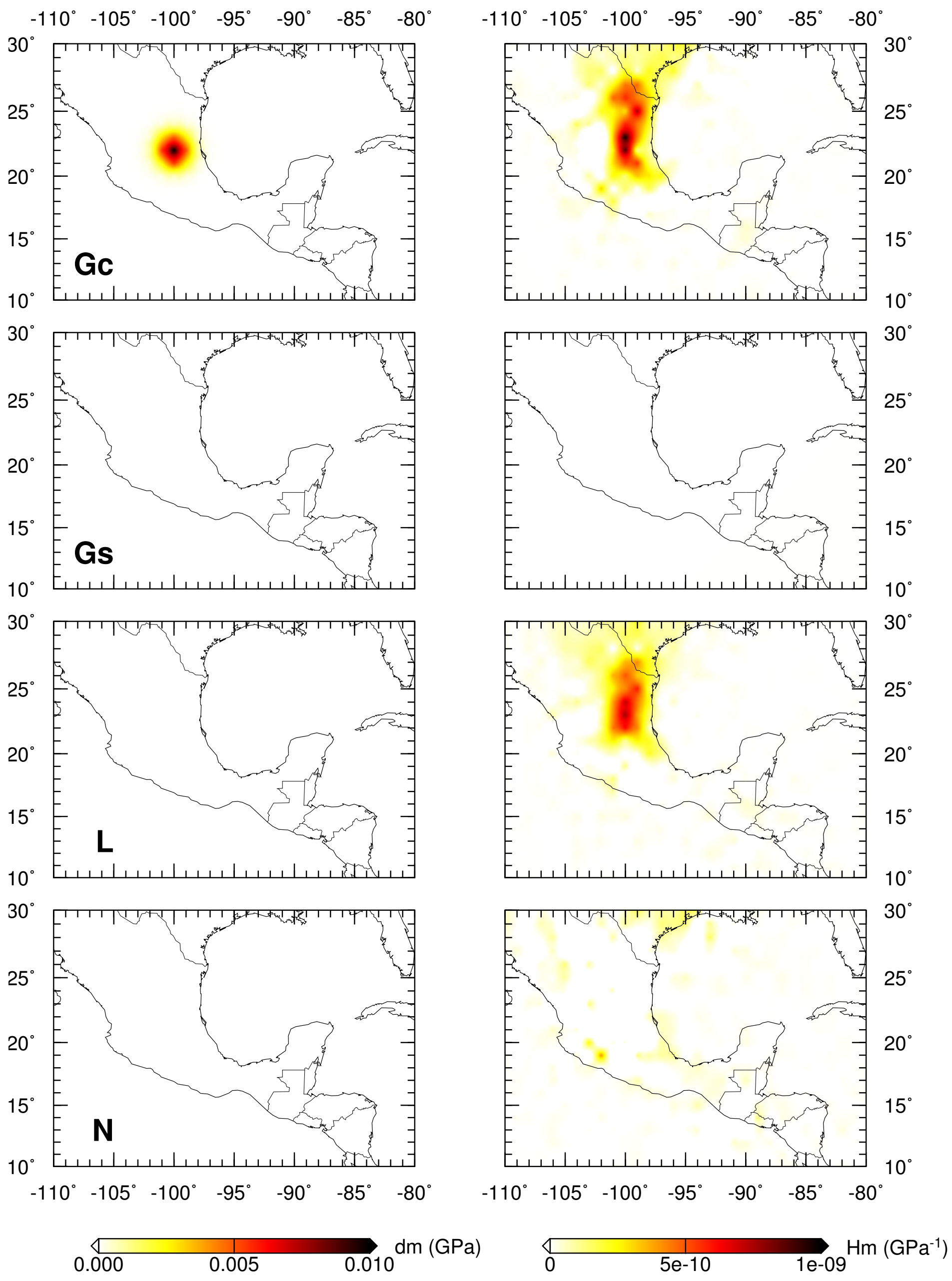

Supplementary Figure 10: The third point-spread function test at 240 km beneath the Rivera Subduction Zone. The same setting as Supplementary Figure 9 except here we perturb G_c instead of G_s.

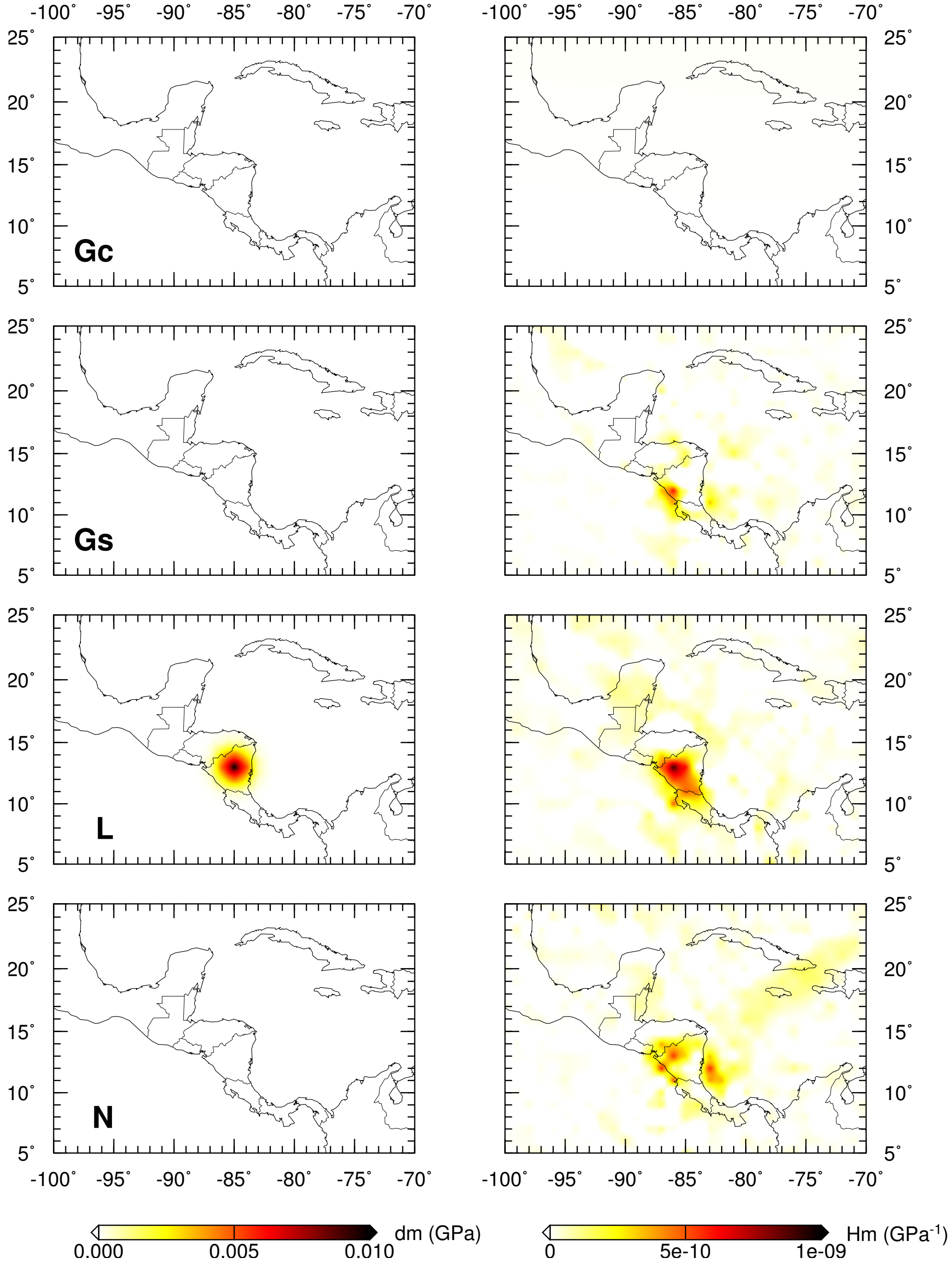

Supplementary Figure 11: The fourth point-spread function test at 240 km beneath the Cocos Subduction Zone. The same setting as Supplementary Figure 9 except here we perturb parameter L.

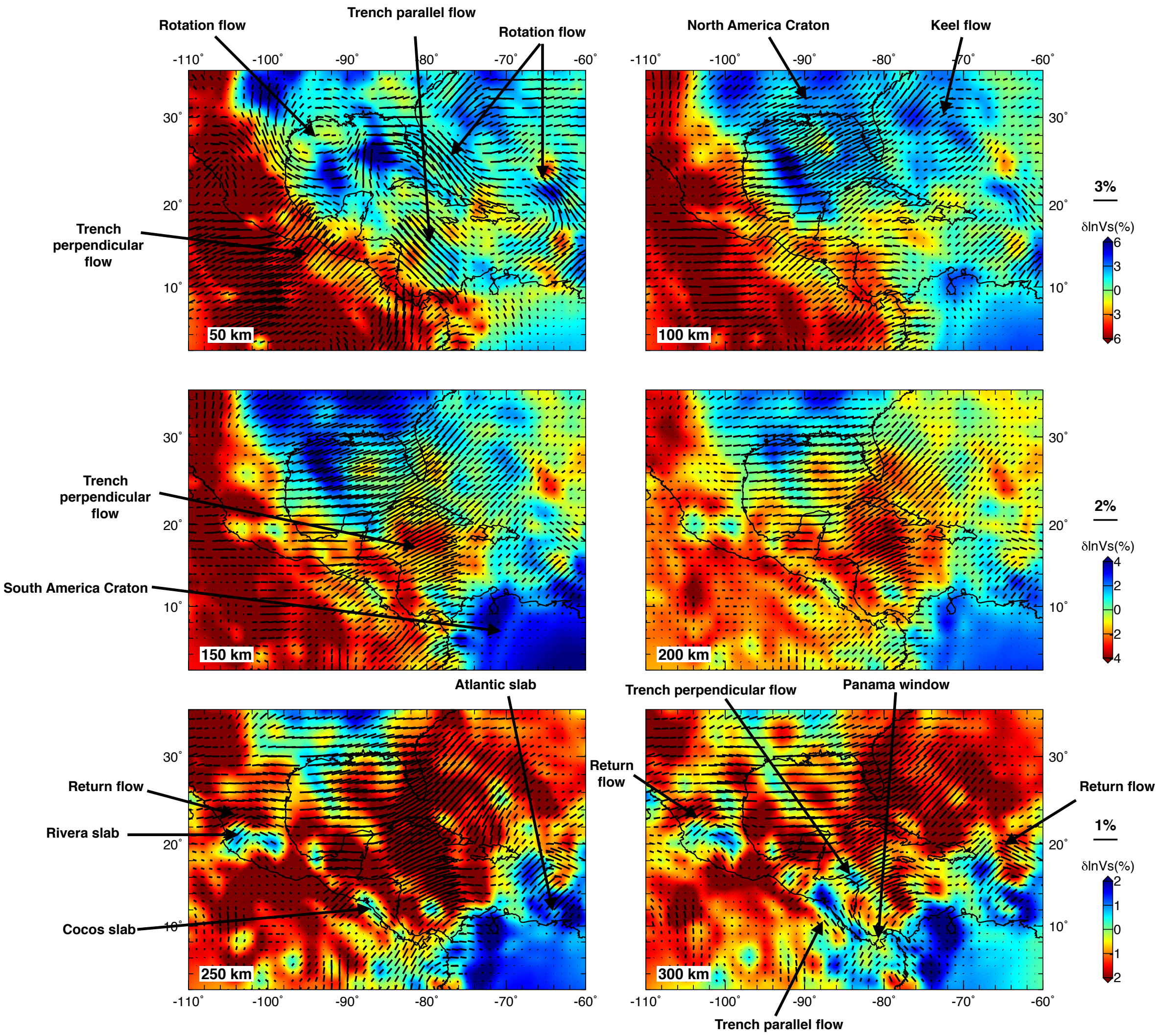

Supplementary Figure 12: Horizontal cross sections of relative perturbations in isotropic shear wavespeed and azimuthal anisotropy at depths ranging from 50 to 300 km in model US_{32}. 1D reference model STW105 [8] is used to calculate the relative wavespeed perturbations. The direction and magnitude of the fast axes are given by the orientation and length of the black bars.

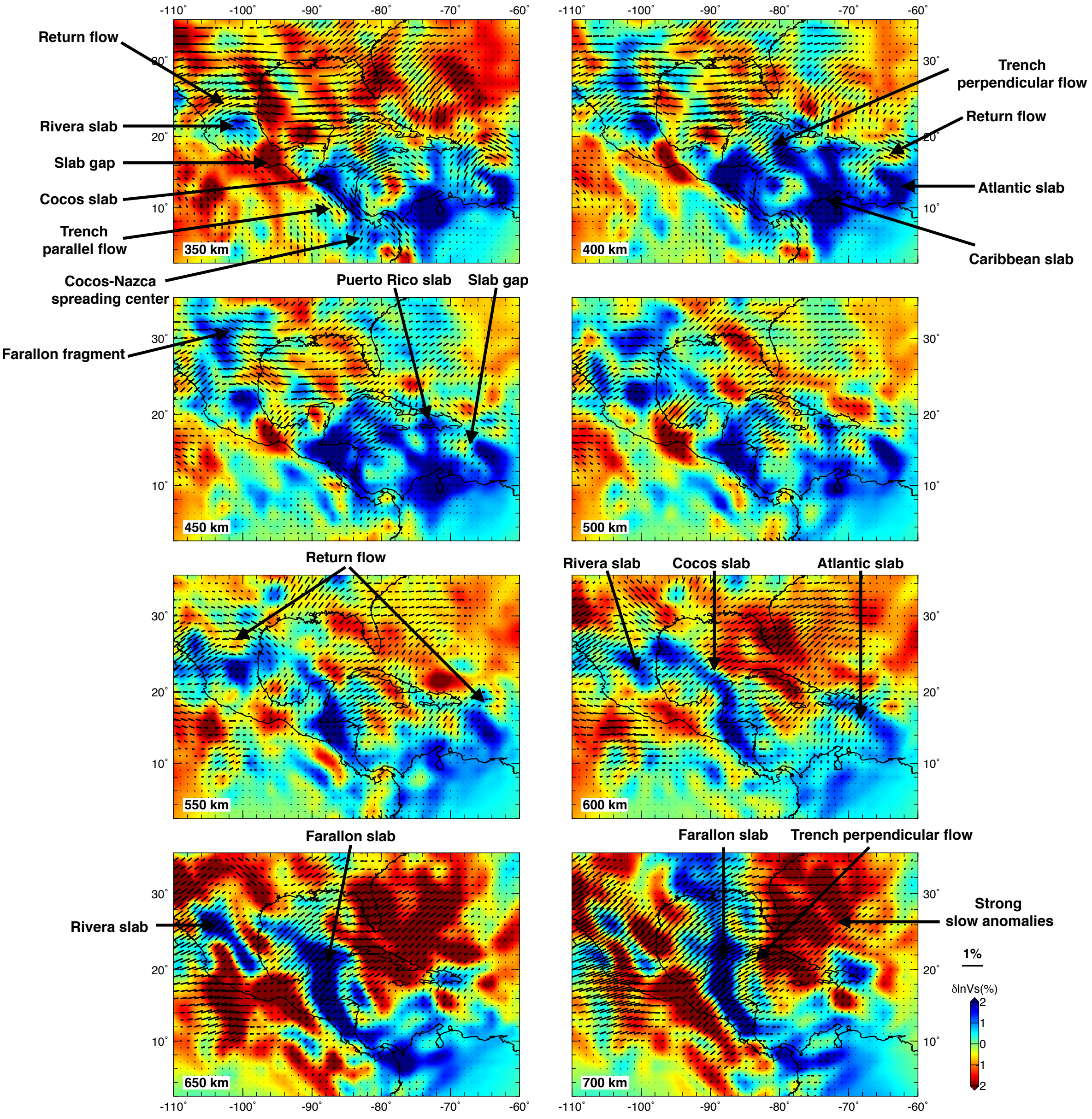

Supplementary Figure 13: The same setting as Supplementary Figure 12 except for depths ranging from 350 to 700 km.

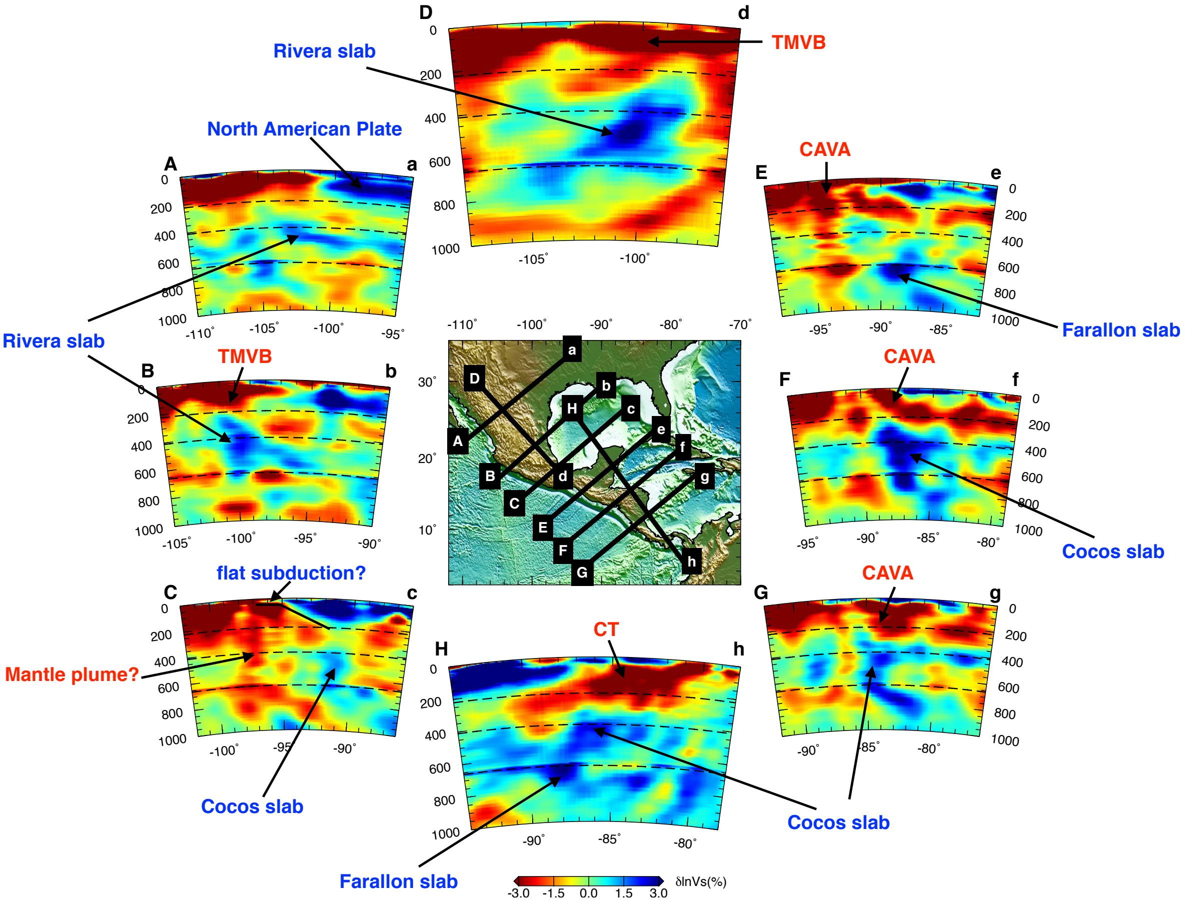

Supplementary Figure 14: Vertical cross sections of relative perturbations in isotropic shear wavespeed along the MAT. The dashed black lines in cross sections denote the 220-, 410- and 660-km discontinuities. CAVA: Central American Volcanic Arc; CT: Cayman Trough; TMVB: Trans-Mexico Volcanic Belt.

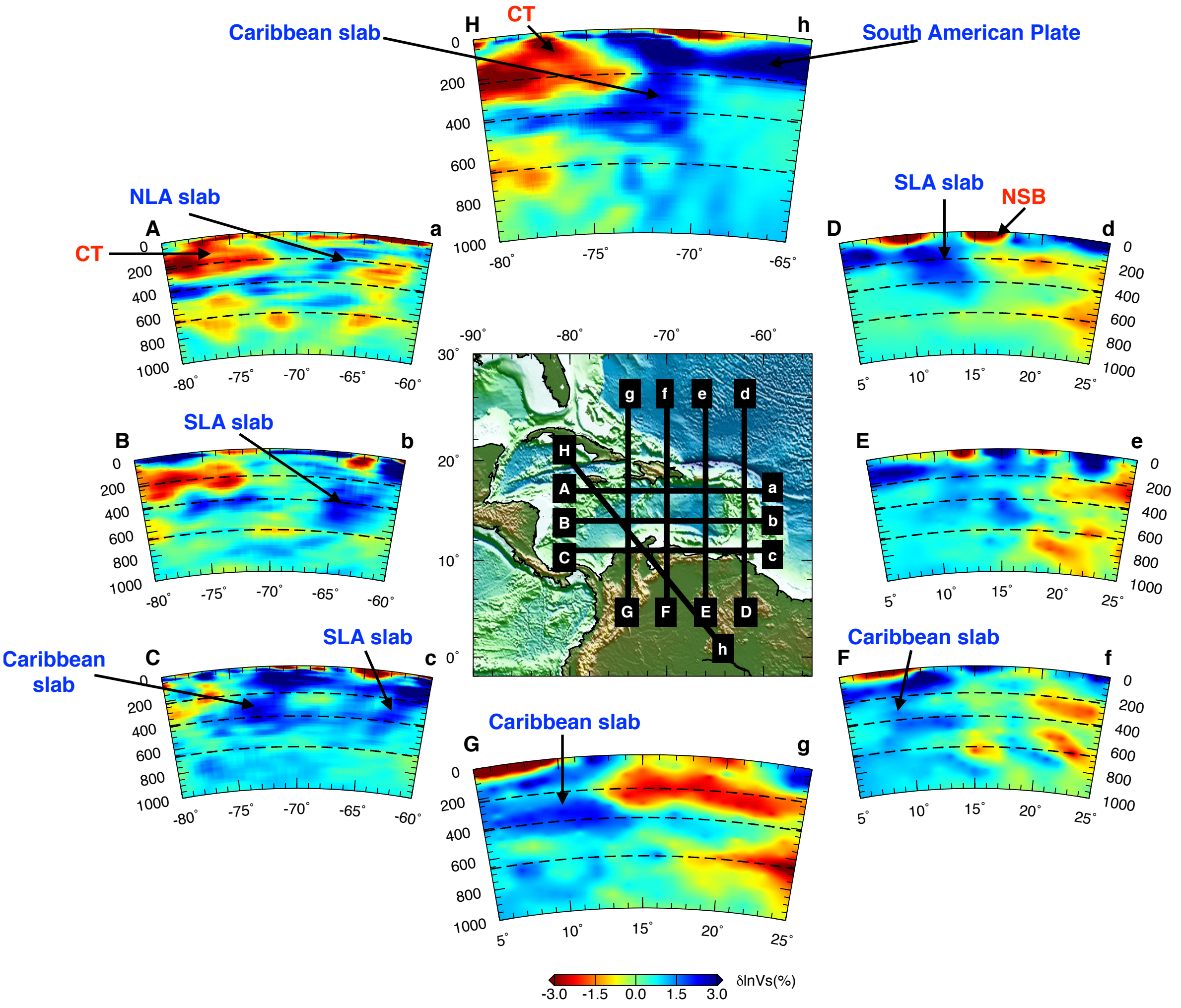

Supplementary Figure 15: Vertical cross sections of relative perturbations in isotropic shear wavespeed for the Caribbean subduction zone. The dashed black lines in cross sections denote the 220-, 410- and 660-km discontinuities. CT: Cayman Trough; NSB: North and South American Plate Boundary; NLA: Northern Lesser Antilles; SLA: Southern Lesser Antilles.

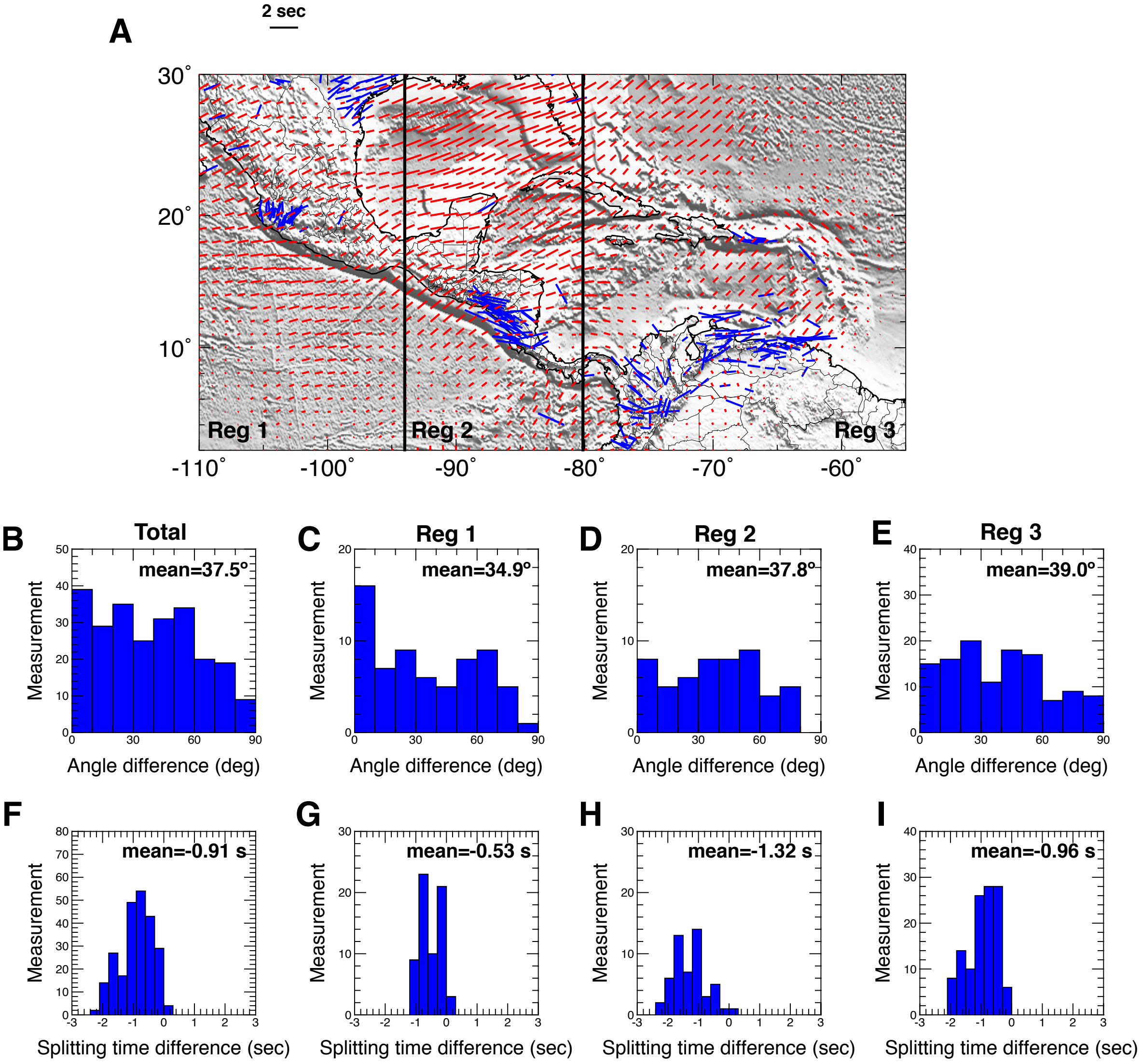

Supplementary Figure 16: Comparisons between predicted and observed shear wave splitting measurements for the study region. Panel A shows the predicted (red) and observed (blue) shear wave splitting measurements. Observed shear wave splitting measurements are collected from http://www-udc.ig.utexas.edu/external/becker/sksdata. Panels B–E show the distributions of the angle differences between observed and predicted SKS measurements for the entire domain, regions 1, 2 and 3, respectively. Panels F–I are the differences on splitting times for these domains. Numbers in the right corner of each histogram represent mean angle and splitting time differences between these two independent measurements.

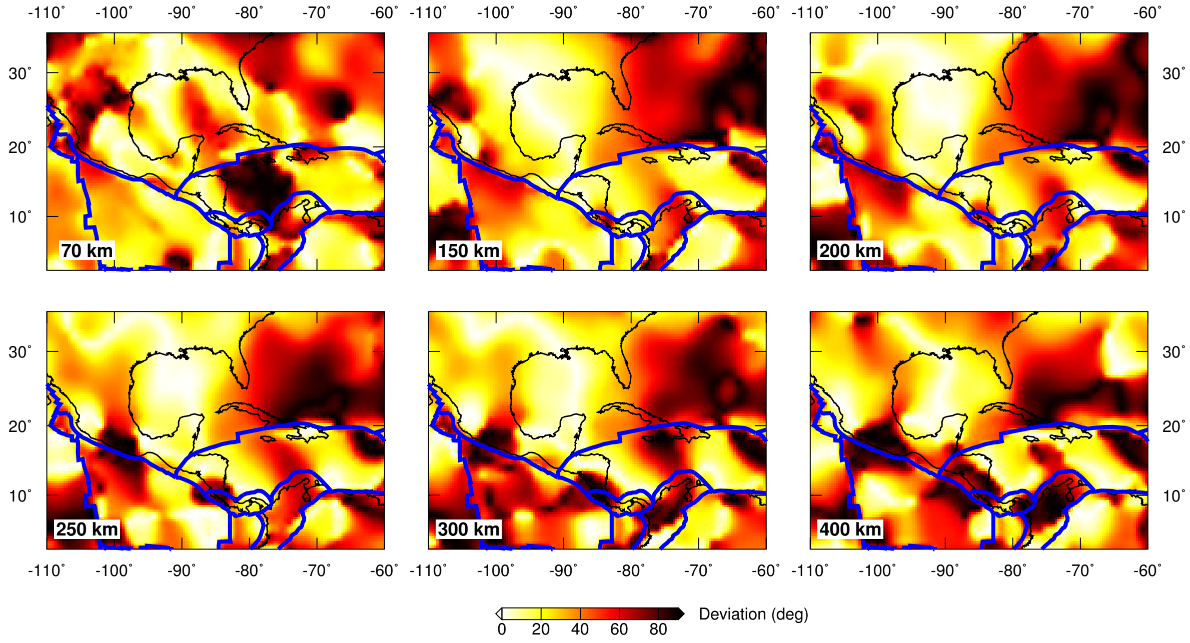

Supplementary Figure 17: The same setting as Figure 8 in the main text, except here we show angle differences between the fast axis orientations in model US_{32} and the plate motion model NUVEL-1A [29] with the no-net rotation reference frame.

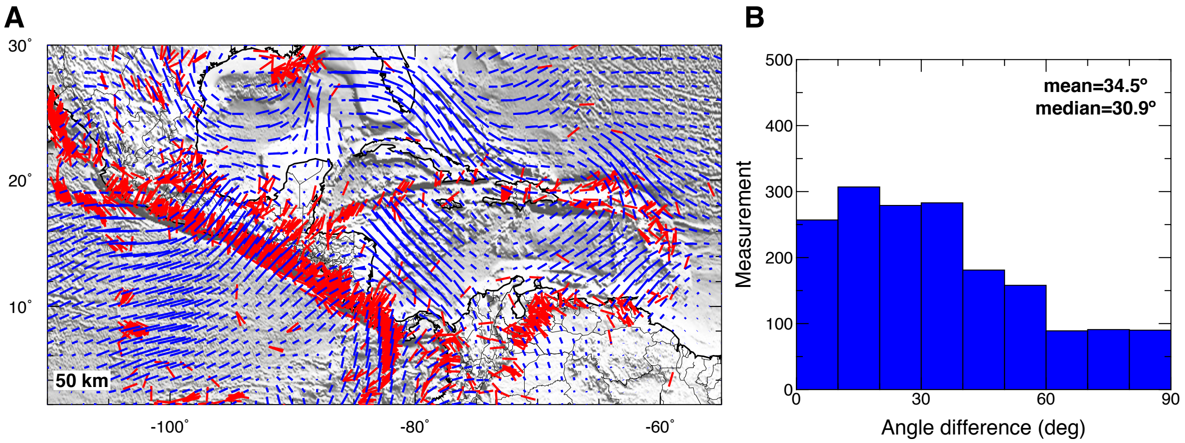

Supplementary Figure 18: Comparisons between the fast axis directions in model US_{32} at a depth of 50 km with maximum horizontal stresses. Panel A compares the fast axis directions (blue) with stress orientations (red) from the global stress state database [16]. Panel B shows the distributions of angle differences between these two independent measurements, and numbers to the right corner denote the mean and median angle differences.

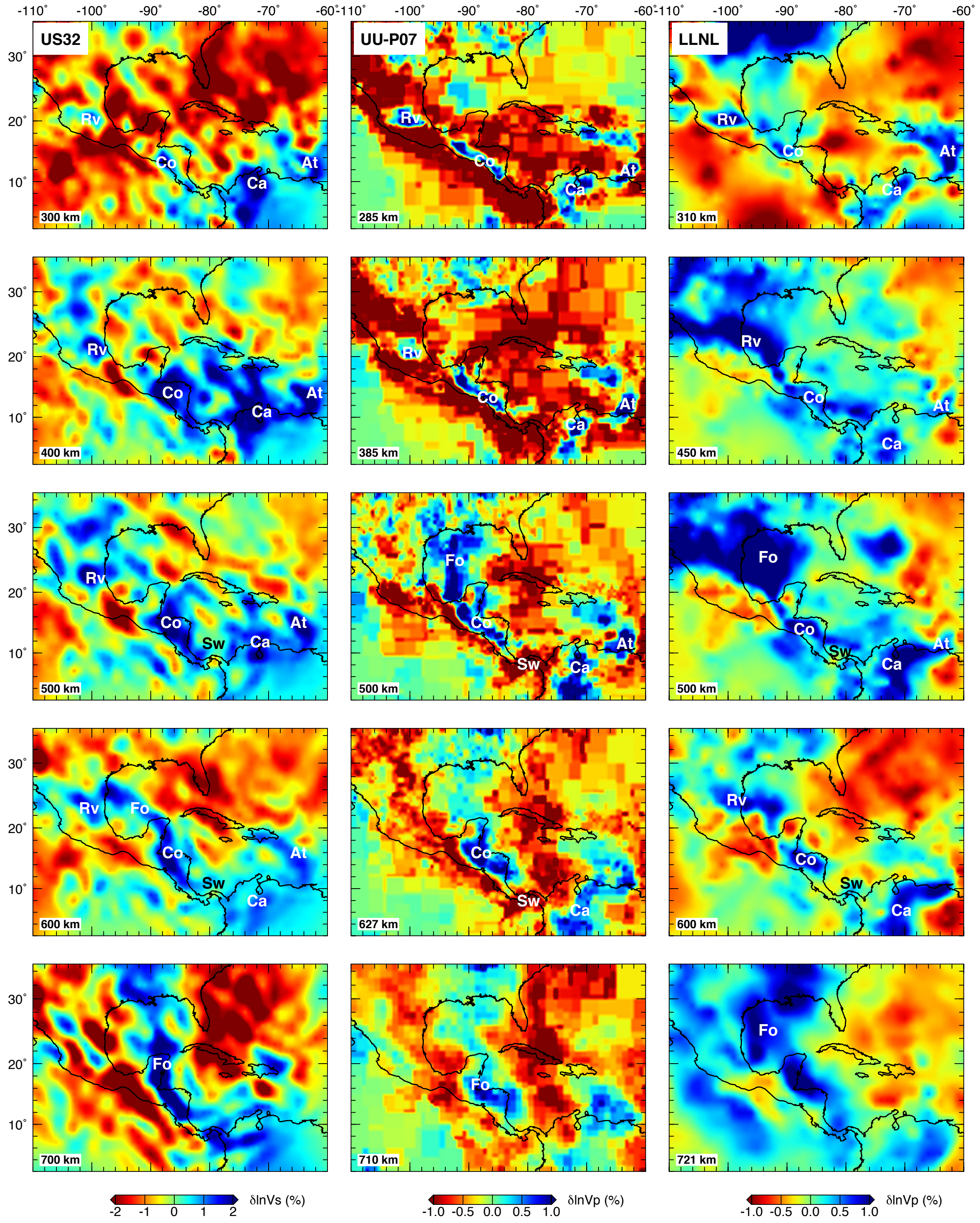

Supplementary Figure 19: Comparisons between the current model US_{32} (left) with two global P wave tomography models: UU-P07 (middle) [17] and LLNL (right) [18] at depths ranging from 300 to 700 km. Rv: Rivera; Co: Cocos; Ca: Caribbean, At: Atlantic; Sw: Slab window; Fo: Farallon.

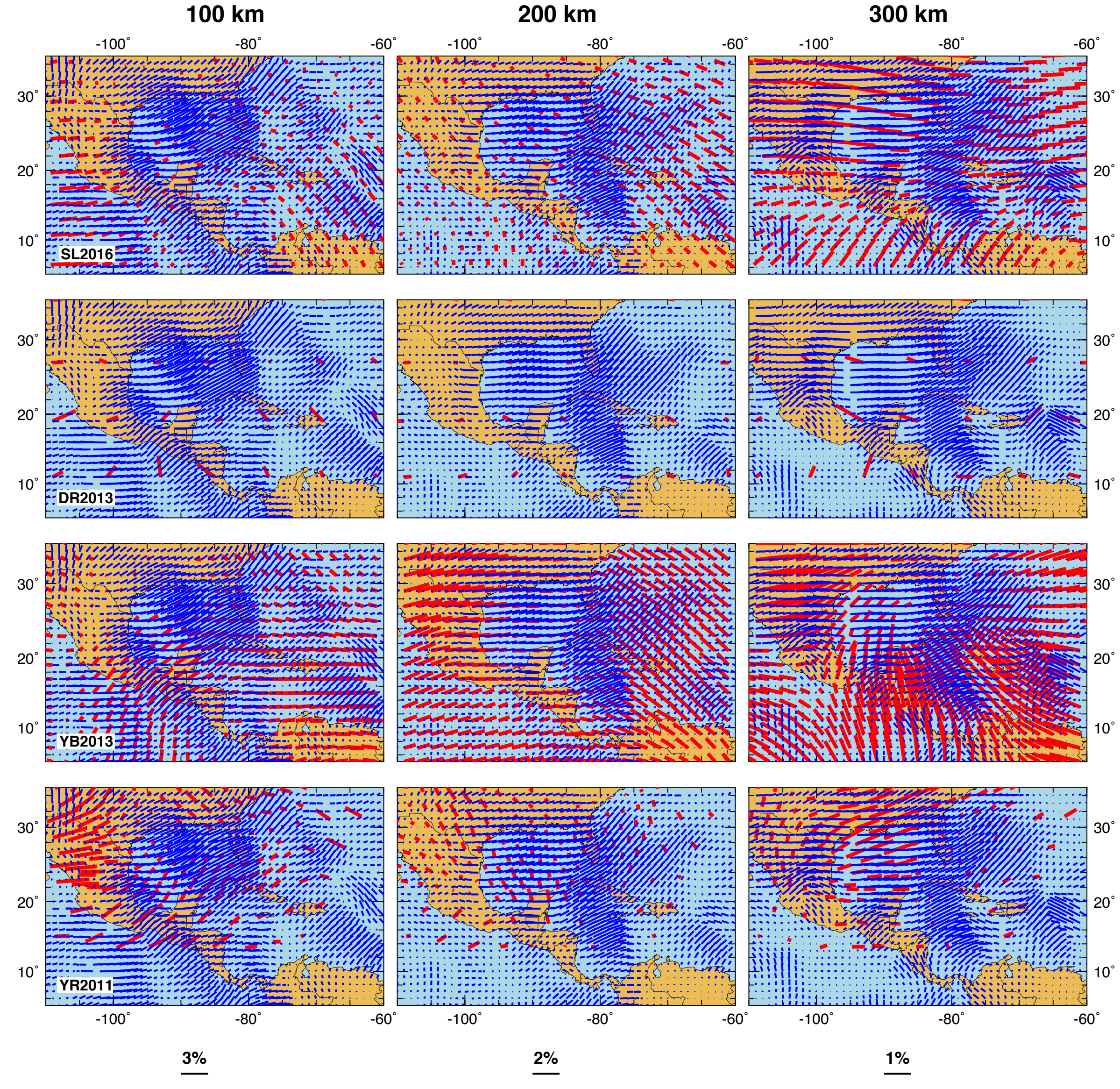

Supplementary Figure 20: Comparisons of the fast axis orientations in model US_{32} (blue) with previous global and continental scale azimuthal anisotropy models (red) at 100, 200 and 300 km (from left to right). From top to bottom are models SL2016 [22], DR2013 [23], YB2013 [24] and YR2011 [25].

Supplementary References

[1] Pardo, M. & Suarez, G. Shape of the subducted Rivera and Cocos plates in southern Mexico: seismic and tectonic implications. *Journal of Geophysical Research* **100**, 12,357–12,373 (1995).

[2] Perez-Campos, X. *et al.* Horizontal subduction and truncation of the Cocos plate beneath central Mexico. *Geophysical Research Letters* **35**, L18303, doi:10.1029/2008GL035127 (2008).

[3] Kim, Y., Clayton, R. & Jackson, J. Geometry and seismic properties of the subducting Cocos plate in central Mexico. *Journal of Geophysical Research* **115**, B06310, doi:10.1029/2009JB006942 (2010).

[4] Kim, Y., Miller, M., Pearce, F. & Clayton, R. Seismic imaing of the Cocos plate subduction zone system in central Mexico. *Geochemistry Geophysics Geosystems* **13**, Q07001, doi:10.1029/2012GC004033 (2012).

[5] van Benthem, S., Govers, R., Spakman, W. & Wortel, R. Tectonic evolution and mantle structure of the Caribbean. *Journal of Geophysical Research* **118**, 3019–3036 (2013).

[6] Harris, C., Miller, M. & Porritt, R. Tomographic imaging of slab segmentation and deformation in the Greater Antilles. *Geochemistry Geophysics Geosystems* **19**, doi/10.1029/2018GC007603 (2018).

[7] Montagner, J., Griot-Pommera, D. & Lava, J. How to relate body wave and surface wave anisotropy? *Journal of Geophysical Research* **105**, 19015–19027 (2000).

[8] Kustowski, B., Ekström, G. & Dziewonski, A. Anisotropic shear-wave velocity structure of the Earth's mantle: A global model. *Journal of Geophysical Research* **113**, B06306, doi:10.1029/2007JB005169 (2008).

[9] Becker, T., Lebedev, S. & Long, M. On the relationship between azimuthal anisotropy from shear wave splitting and surface wave tomography. *Journal of Geophysical Research* **117**, B01306, doi:10.1029/2011JB008705 (2012).

[10] Soto, G. *et al.* Mantle flow in the Rivera-Cocos subduction zone. *Geophysical Journal International* **179**, 1004–1012 (2009).

[11] Hoernle, K. *et al.* Arc-parallel flow in the mantle wedge beneath Costa Rica and Nicaragua. *Nature* **28**, 1094–1098 (2008).

[12] Abt, D. *et al.* Constraints on upper mantle anisotropy surrounding the Cocos slab from SK(K)S splitting. *Journal of Geophysical Research* **115**, B06316, doi:10.1029/2009JB006710 (2010).

[13] Lynner, C. & Long, M. Sub-slab seismic anisotropy and mantle flow beneath the Caribbean and Scotia subduction zones: Effect of slab morphology and kinematics. *Earth and Planetary Science Letters* **361**, 367–378 (2013).

[14] Zoback, M. & Zoback, M. State of stress in the conterminous United States. *Journal of Geophysical Research* **85**, 6113–6156 (1980).

[15] Zoback, M. First- and second-order patterns of stress in the lithosphere: The World Stress Map Project. *Journal of Geophysical Research* **97**, 11,703–11,728 (1992).

[16] Heidbach, O. *et al.* Global crustal stress pattern based on the World Stress Map database release 2008. *Tectonophysics* **482**, 3–15 (2010).

[17] Amaru, M. *Global travel time tomography with 3-D reference models*. Ph.D. thesis, Utrecht University (2007).

[18] Simmons, N., Myers, S., Johannesson, G. & Matzel, E. Llnl-g3dv3: Global P wave tomography model for improved regional and teleseismic travel time prediction. *Journal of Geophysical Research* **117**, doi:10.1029/2012JB009525 (2012).

[19] Grand, S., van der Hilst, R. & Widiyantoro, S. Global seismic tomography: a snapshot of convection in the Earth. *GSA Today* **7**, 1–7 (1997).

[20] Schaeffer, A. & Lebedev, S. Global shear speed structure of the upper mantle and transition zone. *Geophysical Journal International* **194**, 417–449 (2013).

[21] Meschede, M. & Romanowicz, B. Lateral heterogeneity scales in regional and global upper mantle shear velocity models. *Geophysical Journal International* **200**, 1076–1093 (2015).

[22] Schaeffer, A., Lebedev, S. & Becker, T. Azimuthal seismic anisotropy in the Earth's upper mantle and the thickness of tectonic plates. *Geophysical Journal International* **207**, 901–933 (2016).

[23] Debayle, E. & Ricard, Y. Seismic observations of large-scale deformation at the bottom of fast-moving plates. *Earth and Planetary Science Letters* **376**, 165–177 (2013).

[24] Yuan, K. & Beghein, C. Seismic anisotropy changes across upper mantle phase transitions. *Earth and Planetary Science Letters* **374**, 132–144 (2013).

[25] Yuan, H., Romanowicz, B., Fischer, K. & Abt, D. 3-D shear wave radially and azimuthally anisotropic velocity model of the North American upper mantle. *Geophysical Journal International* **184**, 1237–1260 (2011).

[26] Marone, F. & Romanowicz, B. The depth distribution of azimuthal anisotropy in the continental upper mantle. *Nature* **447**, 198–201 (2007).

[27] Laske, G., Masters, G., Ma, Z. & Pasyanos, M. Update on CRUST1.0- a 1-degree global model of Earth's crust. *Geophysical Research Abstracts* **15**, Abstract EGU2013–2658 (2013).

[28] Zhu, H., Komatitsch, D. & Tromp, J. Radial anisotropy of the North American upper mantle based on adjoint tomography with USArray. *Geophysical Journal International* **211**, 349–377 (2017).

[29] Gripp, A. & Gordon, R. Young tracks of hotspots and current plate velocities. *Geophysical Journal International* **150**, 321–361 (2002).
