# Supplementary material for: Seismic evidence for subduction-induced mantle flows underneath Middle America
Source: Nat Commun. 2020 Apr 29;11:2075. doi: 10.1038/s41467-020-15492-6 (PMC7190827; doi:10.1038/s41467-020-15492-6)
Supplement: Supplementary file 2 — Description of Additional Supplementary Files [file 41467_2020_15492_MOESM2_ESM.pdf]

Description of Additional Supplementary Files

File Name: Supplementary Data 1
Description: From left to right columns are longitude (deg), latitude (deg), depth (km), dvs (%), fast axis orientation (deg) and anisotropic magnitude (%).

File Name: Supplementary Movie 1
Description: 3-D iso-surface representation of fast wavespeed bodies with magnitude greater than 1.5%. The north direction points inward. The depths range from the Earth's surface down to 1,000 km. Two white rectangular outlines denote the 410- and 660-km discontinuities.

File Name: Supplementary Movie 2
Description: Shows vertical cross sections moving across model US32 along constant longitudes.

File Name: Supplementary Movie 3
Description: Shows vertical cross sections moving across model US32 along constant latitudes.
